# Screening for substandard and falsified medicines in Nigeria using visual inspection and GPHF-Minilab analysis: lessons learnt for future training of health workers and pharmacy personnel

**DOI:** 10.1080/20523211.2024.2432471

**Published:** 2024-12-09

**Authors:** Micha Lächele, Julia Gabel, Nkiru Sunny-Abarikwu, Rita Ezinwanne Ohazulike, Juliet Ngene, Jane Frances Chioke, Lutz Heide

**Affiliations:** a Pharmaceutical Institute, Eberhard Karls University Tübingen, Tübingen, Germany; b Faith-Based Central Medical Foundation (FBCMF), Enugu, Nigeria

**Keywords:** Substandard medicines, falsified medicines, GPHF-Minilab, thin-layer chromatography, visual inspection, TLCyzer, Nigeria

## Abstract

**Background:**

Substandard and falsified (SF) medicines are a serious threat to public health in low- and middle-income countries (LMICs). Visual inspection of medicines and screening analysis using the Global Pharma Health Fund (GPHF)-Minilab are important in medicine quality surveillance in low-resource settings.

**Methods:**

Recently, 260 medicine samples from Nigeria had been investigated for assay and dissolution according to the United States Pharmacopeia (USP). In the present study, these results were compared to the results of the investigation of the same samples by visual inspection and by GPHF-Minilab analysis by local personnel in Nigeria.

**Results:**

Visual inspection identified many deficiencies of dosage units and packaging information in SF medicines. All four falsified medicines were readily identifiable, primarily from serious spelling errors in the labelling, and from manufacturer names which could not be verified using internet resources. In GPHF-Minilab disintegration testing, two samples did not disintegrate even after 60 min; both were found to fail USP dissolution testing with extreme deviations. Of the 20 samples which deviated in USP assay analysis by more than 20% from the declared API amount, seven (35%) were detected as non-compliant in TLC analysis. Evaluation by TLC image analysis with a recently developed smartphone application (named TLCyzer) increased sensitivity to 62.5% but led to an unacceptably low specificity (75.2%). Additional training of the local personnel improved the results of both TLC analysis and TLCyzer evaluation. Photographs of the visual deficiencies and of the TLC analysis results of the SF medicines are provided as PowerPoint and PDF slides with this publication, for future training courses of pharmacy staff and health workers in LMICs.

**Conclusion:**

Visual inspection, and screening analysis with simple, rapid and inexpensive methods, are important in the surveillance for SF medicines in LMICs. This study provides data on the potential and the limitations of such screenings.

## Background

Substandard and falsified (SF) medicines represent a serious public health risk, especially in low- and middle-income countries (LMICs). The World Health Organization (WHO) has estimated that 10.5% of all medicines in LMICs are substandard or falsified (WHO, 2017a). The number of deaths resulting from the use of SF medicines in only two diseases, malaria and childhood pneumonia, was estimated by the WHO to be between 105.000 and 285.000 annually (WHO, 2017a). If these estimates are correct, SF medicines represent one of the deadliest neglected health problems worldwide.

The WHO recommends a strategy of prevention, detection and response to counter this problem (WHO, 2017b). For the detection of SF medicines, simple and inexpensive screening tools are important, since many LMICs lack the resources for a sufficient number of fully equipped medicine quality control laboratories (Zambrzycki et al., 2021). Among the devices used for medicine quality screening in LMICs, the Global Pharma Health Fund (GPHF)-Minilab (USP, 2020; WHO, 2017a) is the most widely used one. According to the GPHF-Minilab Manual (Jähnke & Dwornik, 2022), the analysis with this tool consists of visual inspection, a simplified disintegration test for solid oral dosage forms, and a qualitative and semi-quantitative analysis of the active pharmaceutical ingredients (APIs) using thin-layer chromatography (TLC). The sensitivity of the GPHF-Minilab in the detection of SF medicines has been discussed with some controversy (Jähnke, 2018; Pan & Ba-Thein, 2018), and additional experimental data on the sensitivity and specificity of this screening tool in field studies are desirable.

We have recently reported results of a study of the quality of medicines collected in Nigeria, carried out in a collaboration between the Faith-Based Central Medical Foundation (FBCMF) in Nigeria and the University of Tübingen in Germany (Gabel et al., 2024). FBCMF is a local church organisation in Enugu, Nigeria, which procures medicines locally and supplies them primarily to faith-based health facilities in Enugu and neighbouring states. In that study, 260 medicine samples were collected by FBCMF in Nigeria and analyzed in the laboratory of the University of Tübingen for assay (=API amount) and for dissolution according to the United States Pharmacopeia (USP). Four of the samples (1.5%) were found to be falsified, not containing the declared API(s). An additional 62 samples (23.8%) were found to be substandard (Gabel et al., 2024).

FBCMF is a member of the Difäm-EPN Minilab Network, which implements medicine quality screening using the GPHF-Minilab (Gnegel et al., 2022). The samples of the above-mentioned study had also been investigated by local personnel of FBCMF, using visual inspection of the samples, simplified disintegration testing and analysis with TLC according to the GPHF-Minilab Manual (Jähnke & Dwornik, 2020). The methods and results of these rapid low-cost screening investigations were not included in the previous publication (Gabel et al., 2024) and are presented here for the first time. The key findings reported in the present publication are:

1. A brief summary of the results of the compendial analysis according to the USP. These have been published in more detail in Gabel et al. (2024).
2. A documentation of the different types of visual deficiencies observed among the 66 SF medicine samples detected in this study.
3. Results of the simplified disintegration testing in comparison with the results of compendial dissolution testing according to the USP.
4. A comparison of the results of the TLC analysis using visual evaluation with the results of compendial assay testing according to the USP, including a calculation of the observed sensitivity and specificity of the TLC analysis in the detection of falsified and of substandard medicines.
5. Results of a first field test of an open-source smartphone application (‘TLCyzer’) for photography and image analysis of TLC plates, which has recently been developed for an improved quantitative evaluation of TLC analyses in medicine quality screening (Hauk et al., 2022).

In the course of the present study, it became apparent that the results of all employed rapid low-cost screening methods (visual inspection, simplified disintegration testing, TLC with visual evaluation, and TLC with quantitative evaluation by image analysis) could be improved by additional training of the personnel carrying out these analyses. So far, only few suitable teaching materials for such training courses are available in the literature (FIP, 2021; Schiavetti et al., 2020; Waffo Tchounga et al., 2023; WHPA, 2011). Therefore, photographs of the visual deficiencies of the investigated SF medicines, and of their TLC analysis, are included in the Supplementary Material of this publication as PowerPoint and PDF slides, which could be useful for future training of pharmacy staff and healthcare workers in LMICs. Furthermore, a checklist for the visual inspection of medicines was developed in this study and is included in the Supplemental Material.

## Methods

### Included and excluded medicines

For each of the 13 APIs investigated in this study, the total number of samples, the number of different brands and different batches, and the number of samples investigated by different analytical methods are presented in Supplemental Table S1. All 260 samples were investigated by compendial assay analysis. Ceftriaxone injections were excluded from compendial dissolution testing and from simplified disintegration testing since these tests only apply for solid oral dosage forms. The seven fluconazole samples collected as capsules were excluded from dissolution testing since no dissolution method is described for them in the USP. For one ciprofloxacin sample not enough dosage units were available for dissolution testing. Disintegration testing and TLC analysis of the fluconazole samples could not be completed in the time available for this study due to the COVID-19 pandemic and to civil unrest in Nigeria. The same applied to the TLC analysis of six ceftriaxone, two atenolol, one ciprofloxacin and one hydrochlorothiazide sample. Furthermore, in the quantitative evaluation of TLC results by image analysis, one ‘chloroquine’ and three ‘cotrimoxazole’ samples were excluded since they were falsified and did not show spots of the declared API(s) which could have been evaluated; in addition, one sustained-release metformin sample was excluded since it showed incomplete extraction of the API in TLC analysis (see Results section), therefore quantitative evaluation of the TLC spot of this sample by image analysis was not meaningful.

### Sample collection

The collection of samples for this study is described in detail in Gabel et al. (2024). The medicines were procured by FBCMF from two types of sources: (a) licensed manufacturers and wholesalers; (b) vendors at the pharmaceutical markets in Onitsha and Enugu with unclear licensing status. A mystery shopper approach was used. Medicines collected from licensed vendors were paid for according to standard FBCMF procedures, and medicines collected from Onitsha and Enugu markets in cash.

### Compendial analysis of medicines

Analysis for assay and dissolution according to the USP 42 was carried out at the Pharmaceutical Institute of the University of Tübingen, Germany, as described by Gabel et al. (2024). For each API, the results of the assay analyses are summarised in Supplemental Table S2.

### Visual inspection of medicine samples

Visual inspection was first carried out by FBCMF staff in Enugu, Nigeria, according to the procedure described in the GPHF-Minilab Manual (Jähnke & Dwornik, 2022). After samples had been sent to Germany, visual inspection was repeated in the laboratory of the University of Tübingen. In both cases, this comprised investigation of dosage units, packaging, and labelling information, and was followed by photographic documentation of the observed deficiencies. Based on the different types of visual deficiencies observed during this study, and on the experience from a final workshop conducted in Enugu (Nigeria) in April/May 2024, the visual inspection checklist shown in Supplemental Table S3 was compiled.

### Simplified disintegration testing

Simplified disintegration testing was carried out by FBCMF in Nigeria as specified in the respective chapter of the GPHF-Minilab Manual (Jähnke & Dwornik, 2022). Six tablets or capsules were immersed in a flask containing 100 mL water at 37°C, and the liquid was stirred or shaken from time to time. Immediate release tablets and capsules are considered as compliant if all six units fully disintegrate within 30 min, while slow-release and enteric-coated products have to withstand this test and must not disintegrate before 30 min.

### TLC analysis with visual evaluation

TLC analysis was carried out by FBCMF in Nigeria, following the monographs of the GPHF-Minilab Manual (Jähnke & Dwornik, 2022) for the respective APIs. As described in that manual, for TLC testing two reference solutions, containing API concentrations corresponding to 100% and 80% of the declared API amount in the respective sample, were applied to the TLC plates for comparison with the sample.

### TLC analysis with quantitative evaluation by image analysis

TLC plates were photographed by FBCMF in Nigeria under standardised conditions, using the photography box described by Hauk et al. (2022). This black wooden box protects the UV_256nm_-illuminated TLC plates from ambient light. It consists of a bottom plate which accommodates the TLC plate, and of a box-shaped lid. The lid has two openings in the sides for insertion of the battery-operated UV lamp supplied with the GPHF-Minilab. A third opening located in the upper side enables capturing a photo of the TLC plate with a smartphone camera. Quantitative analysis of the TLC results was carried out by FBCMF in Nigeria using image analysis of the TLC photographs with the TLCyzer app version 0.4.1 on a Motorola G7 smartphone, as described by Hauk et al. (2022). Using this app, a JPEG photo of approximately 12 megapixel is taken of the TLC plate with the smartphone. After manual positioning of the four corner points of the image to be evaluated, the TLC spots are detected automatically and integrated. For each reference spot, the API amount is manually entered. By fitting a linear function, the app automatically calculates the API amount for each sample spot, expressed as percentage of the declared API amount. The TLCyzer app is available free of charge as GPL open-source software, with instructions for use (Hauk et al., 2022).

### Definitions

For substandard and falsified medicines, the current definitions by the WHO were used (WHO, 2017a): substandard medical products are authorised medical products that fail to meet either their quality standards or their specifications, or both; falsified medical products are products that deliberately/fraudulently misrepresent their identity, composition or source. For TLC analysis with visual evaluation, or with quantitative evaluation by image analysis, sensitivity was defined as the proportion of falsified or substandard samples that the screening test correctly identified as non-compliant (‘fail’). Specificity was defined as the proportion of good quality samples that the screening test correctly identified as compliant (‘pass’). These proportions were calculated as described by Altman and Bland (1994).

## Results

### Compendial analysis of medicines

For all 13 collected types of medicines, the results of assay analysis according to the USP 42 are summarised in Supplemental Table S2. Detailed results for assay and dissolution analysis of all samples are described in Gabel et al. (2024).

### Visual deficiencies observed among the substandard and falsified medicines detected in this study

#### Non-uniformity of dosage units

Visual deficiencies were observed in many of the substandard and falsified medicines identified in this study. Supplemental Fig. S1 depicts two medicine samples which contained within the same bulk container of 1000 tablets three or even seven different kinds of tablets, respectively, with different embossings and different thickness. Chemical analysis revealed that none of these tablets contained any API at all, i.e. both samples represented falsified medicines.

#### Discolouration of dosage units

Supplemental Fig. S2 depicts a sample of chloroquine tablets which contained, besides white tablets, tablets with brown discolouration. When both tablet types were analyzed separately, the white tablets complied with USP specifications for assay, containing 100.6% of the stated API amount, while the brown tablets failed USP specifications, containing only 81.9% of the stated API amount.

Supplemental Fig. S3(A) shows metronidazole tablets with brownish discolouration. Chemical analysis showed that this sample contained only 89.6% of the stated API amount and was therefore non-compliant with USP specifications.

Supplemental Fig. S3(B) depicts hydrochlorothiazide tablets showing black spots. This sample failed USP assay specifications, containing only 87.3% of the stated API amount.

#### Poorly manufactured tablets, with ridges, erosion, cracks, and formation of powder

Supplemental Fig. S4 shows dexamethasone tablets with pronounced ridges, resulting in non-uniform tablet weight and in powder formation in the bulk container. The sample showed an API content of only 79.7% of the declared amount, and an average API dissolution of 67.9% of the declared amount (USP Q value: 80%).

Supplemental Fig. S5 shows tablets of another sample, labelled to contain cotrimoxazole. Lack of physical stability resulted in crumbling of tablets and powder formation. These tablets were falsified and did not contain any cotrimoxazole but 27 mg of paracetamol per tablet.

Supplemental Fig. S6 depicts a sample of metronidazole tablets, collected in a bulk plastic container. These tablets lacked physical stability, resulting in fragmentation of many tablets. The intact tables in this sample complied with specifications, but the fragmentation jeopardised the correct dosage of the medication.

#### Tablets exhibiting a peculiar smell

One sample of metronidazole tablets (Zunagyl®; Zunamediks Pharm. Ltd., Nigeria), supplied in a bulk container, exhibited an unpleasant odour. Chemical analysis showed a gross deviation from assay specifications, i.e. only 48.4% of the stated API amount.

#### Incomplete filling of blisters

Supplemental Fig. S7(A) shows a blister of dexamethasone tablets with one blister compartment left empty, indicating insufficient quality assurance/quality control by the manufacturer. The tablets of this sample were found to contain only 51.3% of the stated amount of the API. Supplemental Fig. S7(B) shows a blister of dexamethasone tablets with one blister compartment containing only half a tablet. This sample failed in dissolution testing, with only 76.0% of the stated API amount dissolving (USP Q value: 80%).

#### Spelling errors in the labelling information

Supplemental Fig. S8 shows two samples labelled as cotrimoxazole (=sulfamethoxazole and trimethoprim) tablets. The first API is misspelled as ‘sulphamethozole’, and the labels have multiple further spelling and capitalisation errors. The samples were falsified and contained neither sulfamethoxazole nor trimethoprim.

Supplemental Fig. S9 depicts a sample of glibenclamide tablets. The name of the API is spelled correctly on the secondary packaging but is misspelled as ‘gilbenclamide’ on the blister. The sample was found to show only 51.2% dissolution of the API (USP Q value: 70%).

Spelling errors occurred not only in the API name but also in other parts of the labelling information. Supplemental Figs S10 and S11 show samples of ciprofloxacin tablets and dexamethasone tablets with the misspellings ‘flim coated’ instead of film coated, and ‘practitoner’ instead of ‘practitioner’. Both samples were found to fail in both assay and dissolution analysis, with assay results of 87.5% and 81.2% (USP limit: 90%), and dissolution results of 73.8% and 69.0% (USP Q value: 80%) of the stated API amount, respectively. Supplemental Fig. S12 shows a package containing 1 × 10 cefuroxime axetil tablets. The front side of the package incorrectly states the content as ‘10 × 1 × 10’ tablets. Chemical analysis showed only 83.2% of the stated API amount (USP limit: 90%).

#### Contradictory labelling information

Supplemental Fig. S13 depicts chloroquine tablets with a different batch number stated on the blister compared to the secondary packaging. This is a strong indication of quality defects or even falsification, and the sample was found to contain only 13.1% of the declared API amount. Notably, another product from the same stated manufacturer has been found previously to contain extremely substandard amounts of the declared APIs (NAFDAC, 2020).

#### Non-verifiable manufacturer names

A powerful but underutilised method to identify falsified medicines is the verification of the labelling information using internet resources. A striking example is given in Supplemental Fig. S14, showing the four falsified medicines which were detected in this study and which did not contain the declared APIs. The names of the manufacturers of these medicines were stated as Leoben Healthcare, Rotac Medical Lab., Citicare Laboratories Ltd, and Weltec Healthcare Ltd, each with a complete street address in Nigeria. However, an internet search revealed that neither websites of these manufactures could be found, nor were they found in the list of manufacturers in Nigeria’s Registered Drug Product Database (NAFDAC, n.d.). Apparently, none of these manufacturers existed. Further, the internet search revealed that the name of one of these manufacturers (‘Citicare Laboratories Ltd.’) had previously been used on a falsified medicine sample (NAFDAC, 2019).

#### Misleading labelling information on the country of origin

Supplemental Fig. S15 depicts a sample of glibenclamide tablets, stating as origin ‘ZLF Pharma UK Limited’, with a street address in London, UK. However, an internet search revealed that this company is not registered as a manufacturer in the UK (Companies House, n.d.), but that ‘ZLF pharmaceutical limited’ [*sic*] is a manufacturer in Wuxi, Jiangsu province, China (ZLF, n.d.). The sample failed dissolution testing with a dissolution of only 64.0% of the declared API (USP Q value: 70%).

#### Non-verifiable registration number

The labels of the four falsified medicines discovered in the present study (Supplemental Fig. S14) showed NAFDAC registration numbers which could not be verified in NAFDAC’s online Registered Drug Product Database (NAFDAC, n.d.). However, that database is still in the process of being completed (Gabel et al., 2024). Therefore, a non-verifiable registration number presently cannot be seen as definitive evidence for falsification.

### Results of simplified disintegration testing

In this study, 212 medicine samples were investigated using the GPHF-Minilab disintegration test by FBCMF staff in Enugu, Nigeria. One of these samples (representing metformin tablets) was a sustained release product. It correctly withstood the disintegration test, and in subsequent dissolution testing according to the USP it complied with the specifications for sustained release metformin tablets. However, it had first been overlooked that this sample was labelled as a sustained release product, and it was incorrectly recorded as failing disintegration testing. This shows the importance of appropriate training on the evaluation of disintegration testing results.

Of the other 211 samples, two (containing chloroquine and atenolol, respectively) were found not to disintegrate at all, even after 60 min. Notably, subsequent dissolution testing according to the USP showed that both failed with extreme deviations, showing dissolution of only 44.7% and 24.0% of the stated API amounts, respectively (USP Q values: 75% and 80%, respectively). Four further samples failed disintegration testing; two of them were reported to show delayed disintegration (35 min), and another two incomplete disintegration. However, these four samples were found to be compliant in subsequent dissolution testing according to the USP.

The other 205 samples passed Minilab disintegration testing. For 43 of these, subsequent USP dissolution testing showed an insufficient amount of dissolved API, lower than the USP specification (Gabel et al., 2024). It should be noted, however, that 29 out of these 43 samples contained an insufficient API amount already in USP assay testing. The other 14 showed a sufficient API amount in assay testing but an insufficient amount of dissolved API in dissolution testing, three of these even with dissolution rates more than 25% below the pharmacopeial threshold.

### Results of TLC analysis with visual evaluation

A total of 228 medicine samples were investigated using TLC by FBCMF in Nigeria. The results exemplify the potential and the limitations of Minilab TLC analysis.

#### TLC identification of medicines which do not contain the declared active ingredient

**Figure 1.**
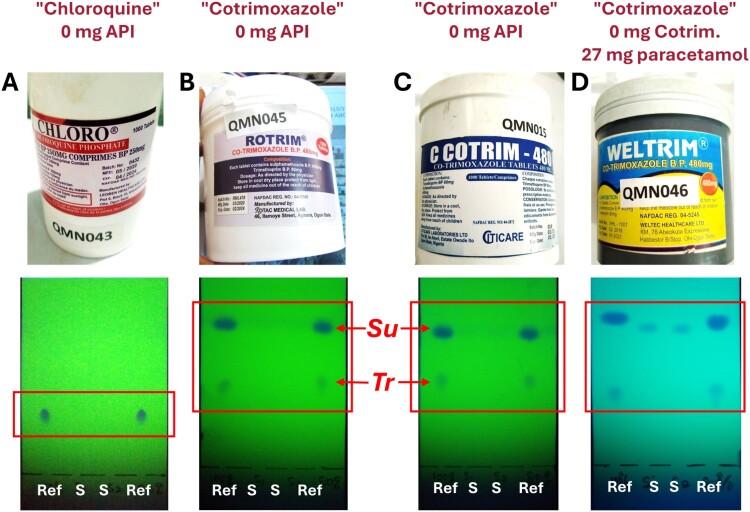
Thin-layer chromatographic identification of medicines which do not contain the declared active pharmaceutical ingredient. Ref = authentic reference substances (left lane: 100%; right lane: 80%); S = sample solutions. *Su* = sulfamethoxazole; *Tr* = trimethoprim. See text (and Methods section) for further explanations.

Four falsified medicine samples were detected in this study. They did not contain the stated API(s), as later proven by HPLC analysis. Photos of these samples and their TLC analyses are shown in Figure 1. Sample A was labelled to contain 250 mg of chloroquine phosphate per tablet. TLC analysis shows the spots of the authentic chloroquine reference substance, but no spots are visible in the two lanes where the sample solution was applied. This proves the absence of TLC-detectable amounts of chloroquine in this sample.

Samples B and C were labelled to contain 480 mg of cotrimoxazole, i.e. a fixed combination of 400 mg of sulfamethoxazole and 80 mg trimethoprim. TLC analysis shows the spots of both APIs for the authentic reference. However, the lanes of the sample solutions show absence of detectable amounts of both APIs.

Sample D was also labelled to contain 480 mg of cotrimoxazole per tablet. As visible from the TLC analysis, the sample solution contains no detectable amounts of sulfamethoxazole or trimethoprim but shows spots of a different substance with an Rf value slightly lower than that of sulfamethoxazole. This substance was subsequently identified in the University of Tübingen as paracetamol (27 mg/tablet), using HPLC, UV spectroscopy and TLC analysis in comparison with authentic paracetamol (Gabel et al., 2024).

Therefore, all four samples which did not contain the declared API were readily identifiable by GPHF-Minilab TLC analysis. The further 224 samples investigated by TLC all proved to contain the declared APIs by subsequent HPLC analysis (Gabel et al., 2024). In two cases, the reported results of their first TLC analyses (depicted in Supplemental Fig. S16) were that the declared APIs cotrimoxazole and cefuroxime axetil were not detected. When the analysis was repeated, as is the standard operation procedure in case of a non-compliant TLC result, the re-tests readily proved the presence of the APIs (Supplemental Fig. S16). Apparently, a handling error had occurred in the first tests. This exemplifies the importance of the instruction to repeat any GPHF-Minilab analysis giving a ‘non-compliant’ result.

#### TLC identification of medicines which contain less than 50% of the declared API amount

**Figure 2.**
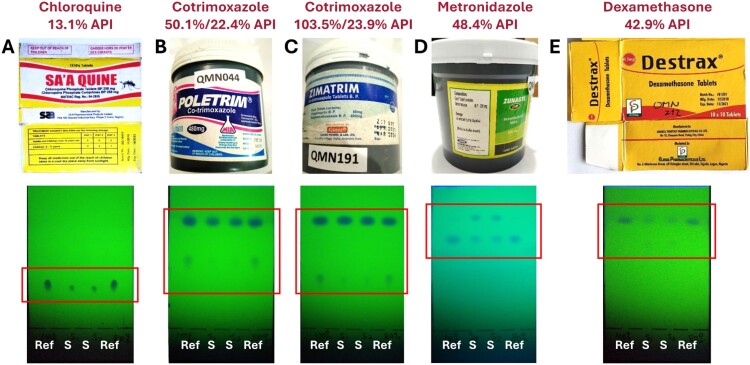
Thin-layer chromatographic analysis of medicines which contain less than 50% of the declared amount of the active pharmaceutical ingredient.

Five medicine samples showed detectable amounts of the declared API(s) in HPLC analysis but less than 50% of the stated amount. These samples are depicted in Figure 2 with their TLC analyses.

Sample A (labelled as chloroquine phosphate tablets 250 mg) contained only 13.1% of the declared API amount. This is clearly visible in the TLC analysis, where the spots of the sample are markedly weaker than those of the authentic reference.

Samples B and C (labelled as cotrimoxazole tablets 480 mg) contained only 22.4% and 23.9% of the declared amount of trimethoprim, respectively. Correspondingly, the spots of trimethoprim in the samples are only faint and weaker than those of the reference. Sample B also contained only 50.1% of the declared amount of sulfamethoxazole. TLC analysis shows that the sulfamethoxazole spots are weaker in the sample than in the reference, but this difference may not be immediately obvious to an untrained investigator.

Sample D (labelled as metronidazole tablets 200 mg) contained only 48.4% of the declared API amount. TLC analysis shows weaker spots for the sample than for the reference and an additional compound with a higher Rf value. This additional compound was also observed in HPLC analysis but remained unidentified in this study.

Sample E (labelled as dexamethasone tablets 0.5 mg) contained only 42.9% of the labelled API amount. TLC analysis shows the insufficient API quantity, as well as faint spots of an additional compound with a lower Rf value. This additional compound was identified in the University of Tübingen as a preservative from the paraben family (Gabel et al., 2024).

Samples A, B and C had been readily identified as non-compliant by FBCMF staff in Enugu, Nigeria, using TLC analysis. However, samples D and E were not reported as non-compliant. This indicates that additional training of analyzer personnel in the semiquantitative evaluation of TLC results may improve the sensitivity of the TLC analysis.

#### TLC identification of medicines which contain between 50% and 80% of the declared API amount

**Figure 3.**
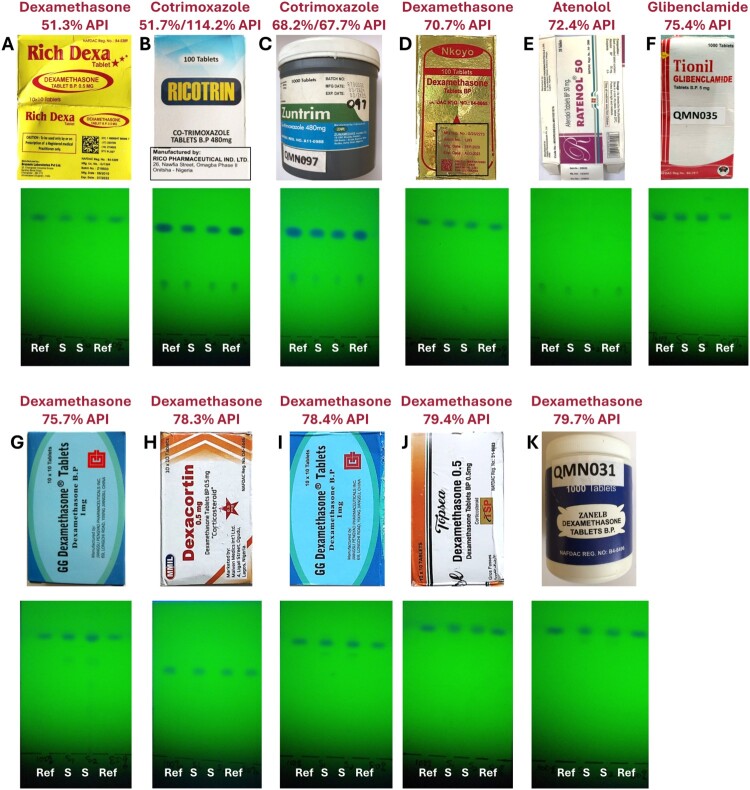
Thin-layer chromatographic analysis of medicines which contain between 50% and 80% of the declared amount of the active pharmaceutical ingredient.

Among the 228 samples investigated using TLC were 11 samples which had API contents between 50 and 80% of the declared amount. Figure 3 shows these samples and the results of their TLC analyses. Samples G and I are tablets of the same brand and batch; sample I had been collected in the first round of sample collection for this study, and sample G seven months later in the second round (Gabel et al., 2024). Seven of the eleven samples depicted in Figure 3 are dexamethasone samples. As described by Gabel et al. (2024), in this study 20 (90.9%) out of the 22 dexamethasone samples were out-of-specification in assay testing; this unusual result was confirmed by a WHO-prequalified medicine quality control laboratory (Gabel et al., 2024).

As expected, the spots of the API(s) were visible in the TLC analyses of all these 11 samples (Figure 3). None of these samples was reported as non-compliant in TLC analysis. It needs to be considered, however, that the seven dexamethasone samples depicted in Figure 3 were labelled to contain 0.5 mg/tablet (five samples) or 1 mg/tablet (two samples). Dexamethasone tablets of higher strength than 0.5 or 1 mg/tablet had not been available at the sampling sites of this study. This strength is below the range of 2–8 mg/tablet described in the GPHF-Minilab monograph for dexamethasone tablets (Jähnke & Dwornik, 2022) and makes precise TLC analysis technically difficult.

For samples A, B and C, a careful evaluation of the TLC analysis (Figure 3) shows that the sample spots are weaker than those of the references. This again suggests that additional training in semiquantitative evaluation of TLC analyses may improve the sensitivity of the TLC detection of SF medicines.

#### TLC identification of substandard medicines which contain more than 80% of the declared API amount

Among the 228 samples investigated using TLC, there were 26 samples which contained more than 80% of the declared API amount, but were still non-compliant in assay testing due to an API content below the lower USP threshold for their respective API (Gabel et al., 2024). The GPHF-Minilab is not designed to detect samples as substandard which contain 80% or more of the declared API amount (Jähnke & Dwornik, 2022), and indeed all these 26 samples were reported as ‘compliant’ in TLC analysis.

In addition, there was one chloroquine tablet sample containing 111% of the declared API amount. This exceeded the upper USP threshold for chloroquine tablets (107%), and therefore this sample was substandard. Unsurprisingly, also this sample was reported as ‘compliant’ in TLC analysis.

#### In-specification samples which were incorrectly reported as ‘non-compliant’ in TLC analysis

**Figure 4.**
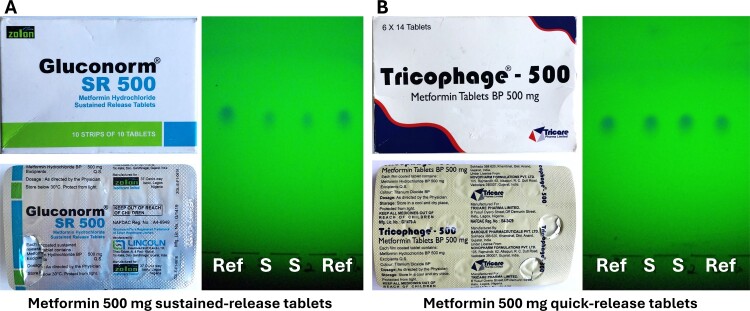
Two in-specification samples which were incorrectly reported as ‘non-compliant’ based on their TLC analysis results. In USP assay analysis, sample A showed 95.1% of the declared amount of the API, and sample B 100.6%.

Only two samples which were in-specification in USP assay analysis had been incorrectly categorised as ‘non-compliant’ by TLC analysis. These samples and their TLC analysis results are depicted in Figure 4.

Sample A was reported by the FBCMF staff to contain less than 50% of the declared amount of the API, estimated from the TLC analysis. Indeed, the TLC analysis depicted in Figure 4(A) is consistent with this estimate. Notably, however, this sample was the only sustained release formulation collected in this study. It appears likely that the sustained release formulation had impeded the extraction of the API, carried out according to the GPHF-Minilab Manual (Jähnke & Dwornik, 2022) (see Discussion section).

Sample B (Figure 4) was reported to contain more than 120% of the declared amount of the API, estimated from the TLC analysis. Possibly, this was an overinterpretation of the slightly stronger spots of the sample as compared to the reference.

#### Improvement of the quality of TLC analysis after additional training of analyzer personnel

**Figure 5.**
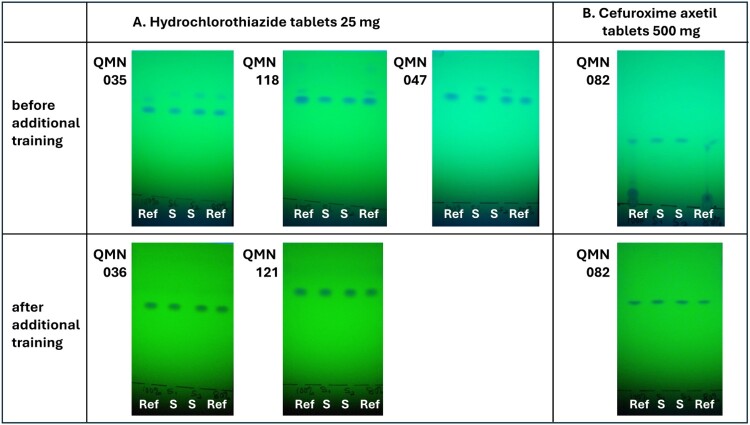
Improvement of the quality of TLC analysis after additional training of analyzer personnel. QMN numbers design different samples of this study.

In October 2022, after 136 (60%) out of 228 TLC analyses of this study had been completed, a two-day refresher training for two staff members of FBCMF was conducted by two staff members of the University of Tübingen in Nairobi, Kenya, during a conference of the Ecumenical Pharmaceutical Network (EPN). Notable improvements were seen in the quality of the TLC analyses after that training. For some APIs such as hydrochlorothiazide, additional spots had been observed in TLC analysis prior to the refresher training, both for samples and for reference compounds (Figure 5(A)). During the training, it was revealed that the Nigerian personnel had followed an outdated procedure of activating the TLC plates (=removing moisture) by placement on the hot plate supplied with the GPHF-Minilab. This activation had been done after the solutions of sample and reference substances were applied to the TLC plates, and apparently led to a partial decomposition of some of the APIs. Decomposition of organic compounds adsorbed to silica gel is a well-known phenomenon (Cai, 2014; Mitchell & Reid, 1931), and is also mentioned in the GPHF-Minilab Manual for several APIs such as β-lactam antibiotics or efavirenz (Jähnke & Dwornik, 2022). The activation procedure was abolished after the refresher training, and subsequent TLC analyses did not show the decomposition anymore (Figure 5(A)).

Prior to the refresher training, some TLC analyses had shown additional spots near the start line, often occurring together with weak or distorted spots of the APIs (Figure 5(B); see Supplemental Fig. S16(B) for another example). The reason for this phenomenon remained unclear. After the appropriate procedures for pipetting, sample application and subsequent drying before TLC plate development had been practiced in the refresher training, this phenomenon was not observed anymore.

#### Sensitivity and specificity of TLC analysis for the identification of substandard and falsified medicines

**Figure 6.**
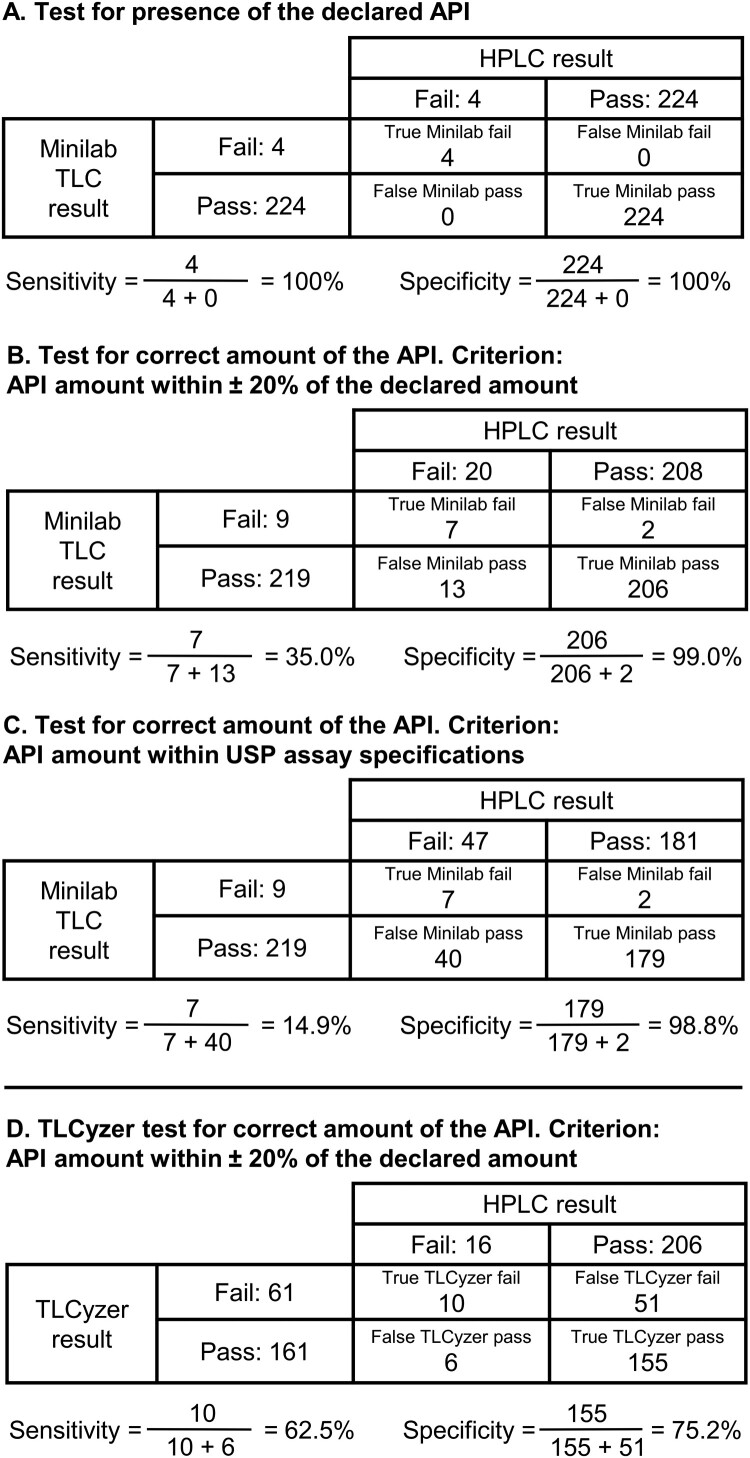
(**A–C**) Sensitivity and specificity of TLC analysis with visual evaluation for the identification of substandard and falsified medicines (*n* = 228 samples). (**D**) Sensitivity and specificity of TLC analysis with image analysis evaluation using the TLCyzer smartphone application (Hauk et al., 2022) (*n* = 222 samples). Sensitivity and specificity were defined as explained in the Methods section.

All four falsified samples, not containing the declared API(s), were readily identifiable by TLC analysis, resulting in 100% sensitivity for this (very small) group of samples (Figure 6(A)). TLC analysis also correctly identified all 224 samples which did contain the declared API, resulting in 100% specificity, provided that the standard operation procedure to repeat GPHF-Minilab analyses with a ‘non-compliant’ result was followed. As mentioned above, in the first TLC analysis two samples had been incorrectly reported as not containing the declared API, resulting in a specificity of 99.1% from this first analysis alone.

Besides the four samples not containing the declared API(s), 16 further samples contained less than 80% of the stated amount of declared API(s). Seven of all these 20 samples, representing the most extreme deviations, were detected by TLC analysis, while 13 were not, resulting in a sensitivity of 35%. Out of the 208 samples deviating by less than 20% from the declared API amount, 206 were correctly identified as compliant with GPHF-Minilab criteria (Jähnke & Dwornik, 2022), resulting in a specificity of 99.0% (Figure 6(B)).

The USP specifies more narrow compliance limits for the content of the APIs compared to the GPHF-Minilab criteria. Within the medicines in this study, these USP limits ranged from 95 to 105% of the declared amount for metformin tablets to 90–115% for ceftriaxone injections (Gabel et al., 2024). Among all 228 samples investigated by TLC, a total of 47 contained API amounts deviating from USP assay specifications. Only seven of these, again representing the most extreme deviations, were detected by TLC analysis, while 40 were not, resulting in an (expectedly low) sensitivity of 14.9%. Out of the 181 samples containing an API amount within USP specifications, 179 were identified as compliant by TLC analysis, resulting in a specificity of 98.8% (Figure 6(C)).

### Results of TLC analysis with quantitative evaluation by image analysis using a smartphone application

222 samples were evaluated by FBCMF in Nigeria using TLC photography and image analysis with the recently developed open-source TLCyzer smartphone app (Hauk et al., 2022). The overall result is shown in Figure 6(D). For the detection of samples deviating by more than 20% from the stated API amount, evaluation with the TLCyzer app showed a sensitivity of 62.5%, exceeding the 35.0% obtained with visual evaluation. However, the specificity was unacceptably low (75.2%), much lower than the 99.0% obtained with visual evaluation.

Among the 222 samples, the recovery rate of the TLCyzer quantification of the API amount (i.e. the TLCyzer result expressed as % of the HPLC assay result) showed a median value of 98.8%, and a mean value of 102.0%. This proves the absence of a relevant systematic error in the TLCyzer quantification. However, there was a high random variability. Among all 222 samples, the relative standard deviation (RSD) between TLCyzer result and HPLC assay result showed a median value of 8.4% and a mean value of even 16.4%. As apparent from this difference between median and mean, there were many samples with extremely high deviations between TLCyzer and HPLC results. As mentioned above, a refresher training in the handling of TLC analyses was carried out after approximately 60% of the TLC and TLCyzer analyses of this study had been completed. This also included re-training in the use of the TLCyzer app. Among the 131 samples investigated before this training, the RSD had been 10.3% (median) and even 22.0% (mean). Among the 91 samples investigated after the training, the RSD was 7.0% (median) and 8.3% (mean). As shown by the much lower difference between median and mean, after the training there were much fewer samples with extremely high deviations between TLCyzer and HPLC results. While Figure 6(D) shows an overall 75.2% specificity for the TLCyzer evaluation of all 222 samples, this value was 90.8% for the 91 samples investigated after the training. However, also 90.8% specificity is still unacceptably low for medicine quality screening. Therefore, the present study showed that the TLCyzer app is not fit for deployment in the field at present. Possible reasons which may have contributed to this result include:

1. A reproducible, complete extraction of the APIs from the sample and the reference tablets may not be achievable in the field following the GPHF-Minilab procedure (Jähnke & Dwornik, 2022): tablets were wrapped in aluminum foil, crushed with a pestle, transferred from the aluminum foil to a vial, and extracted with the respective solvent by manual shaking for three minutes. In contrast, for compendial testing tablets were crushed to a fine powder in a mortar and extracted with the respective solvent for 10 min in an ultrasonic bath. Figure 4(A) strongly suggests incomplete extraction of the sample tablets for TLC analyses. A possible solution may be the use of inexpensive general-purpose ultrasonic baths for API extraction prior to TLC analysis.
2. Under field conditions, a sufficiently precise handling of the TLC analyses required for quantitative TLCyzer analysis may not be achievable.
3. Only photos of TLC analyses with very clean backgrounds, like the three photos given in the supplementary information of Hauk et al. (2022), could be evaluated with the TLCyzer app with good repeatability. In contrast, the TLCyzer evaluation of the photos in the present study showed poor repeatability, also when the TLC photos were re-evaluated in the University of Tübingen, and even within different photos of the same TLC plate. The use of smartphones with more powerful cameras (OnePlus 9Pro and Huawei P30, instead of Motorola G7) made this problem worse, not better (data not shown). Possibly, the image processing algorithms of the smartphones (Morikawa et al., 2021) interfered with the quantitative evaluation of the.jpg images of the TLC plates. A solution may be the development of an image analysis application which uses uncompressed and unprocessed images in .raw format.

## Discussion

The present study, based on the investigation of more than 200 medicine samples collected in Nigeria, provides experience from the use of the different screening methods for the detection of SF medicines described in the GPHF-Minilab Manual (Jähnke & Dwornik, 2022), i.e. visual inspection of medicines, a simplified disintegration testing procedure, and TLC analysis. The present publication also provides photos of samples of SF medicines, and of their TLC analysis, as PowerPoint and PDF slides for the training of pharmacy and healthcare staff (Supplemental Figs S1–S21).

Visual inspection of the SF samples of this study showed different types of deficiencies of dosage units, packaging and labelling information. Notably, all four falsified medicines (Supplemental Fig. S14) were readily identifiable by visual inspection. Principal clues for the falsification were given by serious spelling errors of the stated API(s) in the labelling, by non-verifiability of the manufacturers’ names and locations in an internet search, and in some cases by the presence of different types of tablets in the same package.

Not all recognisable visual deficiencies were recorded by the FBCMF staff in Nigeria in this study. The initial training for this study had focused more strongly on TLC analysis than on visual inspection, and this may have resulted in less time of the investigators spent on visual inspection. A similar observation has been made previously (Caillet et al., 2021). Therefore, in a final research dissemination workshop conducted in Enugu, Nigeria, in April/May 2024, also a training in visual inspection was included. For this purpose, a visual inspection checklist was developed, modified from Schiavetti et al. (2020). Based on experience during the training, this checklist was further improved, and the final version is provided as Supplemental Table S3, with explanations of the modifications introduced into the template of Schiavetti et al. (2020). This checklist may be useful in trainings for pharmacy personnel and healthcare workers.

In such training courses it should be mentioned that visual deficiencies, especially minor spelling errors, occur also in medicines which comply with pharmacopeial specifications in chemical analysis. However, medicines with visual deficiencies should be preferentially selected for chemical investigation.

GPHF-Minilab disintegration testing and subsequent compendial dissolution testing suggested that a complete failure of disintegration even after 60 min predicts extreme failure in dissolution testing. Such samples should be quarantined to prevent them from reaching the patient, even before the result of a confirmatory compendial analysis is available. Conversely, compliance of a sample in the GPHF-Minilab disintegration test is not sufficient to predict compliance in dissolution testing. Also, the QAMSA study (WHO, 2011) had found a low sensitivity of the GPHF-Minilab disintegration test for the prediction of non-compliance in dissolution; but when GPHF-Minilab disintegration testing failed, the probability of dissolution failure was high (75%).

The present study confirmed that TLC analysis with the GPHF-Minilab is suitable to reliably identify falsified medicines not containing the declared API(s) (Figure 1). Furthermore, Figure 2 shows that it is suitable to detect medicines containing less than 50% of the declared API amount. However, in the present study only three out of five medicines containing less than 50% had been correctly reported as non-compliant, suggesting a need for further improvements in the semiquantitative evaluation of TLC results, e.g. by continuous training and supervision, and/or by a rule that each TLC plate must be evaluated by two investigators. Notably, the results of the TLC analyses in the present study were much better than those by Risha et al. (2006) who reported that only 3 out of 28 samples containing 40% of the declared API amount were reported as ‘non-compliant’ in GPHF-Minilab testing. After additional training, however, the detection rate increased to 19 out of 27 samples (Risha et al., 2006).

In the present study, none of the nine samples containing between 50 and 80% of the declared API amount were reported as non-compliant in TLC analysis. While the insufficient content of the three samples depicted in Figure 3(A–C) may well be recognisable from their TLC analyses after additional training of the analyzer personnel, the insufficient content of the eight samples depicted in Figure 3(D–K) is not recognisable from the depicted TLC analyses, even for a well-trained investigator.

For future training courses, the TLC results depicted in Figures 1–5 may be useful, and are therefore also provided as PowerPoint and PDF slides in Supplemental Figs S17–S21.

Overall, in this study the sensitivity of TLC analysis to detect samples deviating by more than 20% from the declared API amount resulted as 35%. This is somewhat lower than the sensitivity of 43% observed in a previous study of our group, carried out with similar methodology (Schäfermann et al., 2020). The lower sensitivity in the present study may be related to the high number of substandard dexamethasone samples with strengths of 0.5 or 1 mg per tablet, which is below the range of 2–8 mg per tablet defined in the GPHF-Minilab Manual (Jähnke & Dwornik, 2022). This very low dosage makes precise sample preparation for TLC analysis technically difficult. In the QAMSA study (WHO, 2011), investigating antimalarial medicines, 75% of the medicine samples deviating by more than 20% from the declared API amount had been correctly reported as non-compliant in GPHF-Minilab TLC analysis. These analyses were carried out by personnel of the national medicine regulatory agencies of the involved countries.

Simple, inexpensive screening methods with good sensitivity for the detection of medicine samples with incorrect API amounts would be very helpful, but are currently not available for field use (Zambrzycki et al., 2021). The present study included a first field test of the TLCyzer smartphone app (Hauk et al., 2022) for the quantitative evaluation of TLC analysis results. Unfortunately, a very high random variability of the results was observed. Though this variability was somewhat reduced after additional training of the analyzer personnel, the specificity of this method in the field remained unacceptably low. Therefore, the present study showed that the TLCyzer app is not fit for deployment in the field at present.

## Conclusion

The present paper provides experience from the use of the different screening methods described in the GPHF-Minilab Manual for the detection of SF medicines. It includes data on the sensitivity and specificity of TLC analysis, both in the detection of falsified medicines which do not contain the declared API, and in the detection of substandard medicines with different degrees of severity of their quality deviations. The limited sensitivity of these methods observed in the present study may partly be overcome in the future by additional training, as positive effects of additional training could be clearly demonstrated. For this reason, this publication also provides teaching materials for such training courses of pharmacy staff and healthcare workers in LMICs. Rapid, simple and inexpensive screening methods are important in medicine quality surveillance in LMICs, and the further development of existing methods like the GPHF-Minilab, as well as the development of new methods, are important priorities in global health research.

## Supplementary Material

Supplementary_Table_S3_Visual_inspection_checklist_Revised.pdf

Supplementary_Figures_S1_to_S21_revised.pptx

Supplementary_Table_S1_Overview_of_medicines_investigated_Revised.pdf

Supplementary_Table_S2_Summary of the results of compendial assay analysis_Revised.pdf

## Data Availability

All data generated or analyzed during this study are included either in this published article and its Supplemental Material, or in the preceding publication (Gabel et al., 2024) and its supplementary information files.

## References

[CIT0001] Altman, D. G., & Bland, J. M. (1994). Diagnostic tests 1: Sensitivity and specificity. *British Medical Journal*, *308*(6943), 1552–1552. 10.1136/bmj.308.6943.1552 8019315 PMC2540489

[CIT0002] Cai, L. (2014). Thin layer chromatography. *Current Protocols Essential Laboratory Techniques*, *8*, 6.3.1–6.3.18.

[CIT0003] Caillet, C., Vickers, S., Vidhamaly, V., Boutsamay, K., Boupha, P., Zambrzycki, S., Luangasanatip, N., Lubell, Y., Fernandez, F. M., & Newton, P. N. (2021). Evaluation of portable devices for medicine quality screening: Lessons learnt, recommendations for implementation, and future priorities. *PLoS Medicine*, *18*(9), e1003747. 10.1371/journal.pmed.1003747 34591861 PMC8483386

[CIT0004] Companies House. (n.d.). *ZLF Pharma UK Limited. Company number 11481686*. Retrieved August 2, 2024, from https://find-and-update.company-information.service.gov.uk/company/11481686/more

[CIT0005] FIP. (2021). *Curriculum for pharmacy students on substandard and falsified medicines: Curriculum guide and competency framework*. International Pharmaceutical Federation. https://www.fip.org/file/4917

[CIT0006] Gabel, J., Lächele, M., Sander, K., Gnegel, G., Sunny-Abarikwu, N., Ezinwanne Ohazulike, R., Ngene, J., Chioke, J. F., Häfele-Abah, C., & Heide, L. (2024). Quality of essential medicines from different sources in Enugu and Anambra, Nigeria. *The American Journal of Tropical Medicine and Hygiene*, *111*(1), 179–195. 10.4269/ajtmh.23-0837 38740019 PMC11229646

[CIT0007] Gnegel, G., Häfele-Abah, C., Neci, R., Alladjaba, M., Lächele, M., Grace, N., Djekadoum, N., Gounouman, J. B., Mpawenimana, S., Muziganyi, E., Mukamanzi, A., Zawadi, J. C., Cletus, T. A., Ngah, N. E., Chakraborty, B., Mutombo, G. M., Chioke, S. J. F., Okpan, E., Ngene, J., … Heide, L. (2022). Surveillance for substandard and falsified medicines by local faith-based organizations in 13 low- and middle-income countries using the GPHF Minilab. *Scientific Reports*, *12*(1), 13095. 10.1038/s41598-022-17123-0 35908047 PMC9338985

[CIT0008] Hauk, C., Boss, M., Gabel, J., Schafermann, S., Lensch, H. P. A., & Heide, L. (2022). An open-source smartphone app for the quantitative evaluation of thin-layer chromatographic analyses in medicine quality screening. *Scientific Reports*, *12*(1), 13433. 10.1038/s41598-022-17527-y 35927306 PMC9352711

[CIT0009] Jähnke, R. W. O. (2018). Letter to the editor on previously published GPHF-Minilab assessment. *The American Journal of Tropical Medicine and Hygiene*, *98*(6), 1880. 10.4269/ajtmh.18-0127a 29877172 PMC6086185

[CIT0010] Jähnke, R. W. O., & Dwornik, K. (2020). *A concise quality control guide on essential drugs and other medicines. Physical testing and thin-layer chromatography*. Global Pharma Health Fund e.V.

[CIT0011] Jähnke, R. W. O., & Dwornik, K. (2022). *A concise quality control guide on essential drugs and other medicines. Physical testing and thin-layer chromatography. Review and extension 2022*. Global Pharma Health Fund e.V. (GPHF).

[CIT0012] Mitchell, J. A., & Reid, E. E. (1931). The decompositon of ketones in the presence of silica gel. *Journal of the American Chemical Society*, *53*(1), 330–337. 10.1021/ja01352a049

[CIT0013] Morikawa, C., Kobayashi, M., Satoh, M., Kuroda, Y., Inomata, T., Matsuo, H., Miura, T., & Hilaga, M. (2021). Image and video processing on mobile devices: A survey. *The Visual Computer*, *37*(12), 2931–2949. 10.1007/s00371-021-02200-8 34177023 PMC8215099

[CIT0014] NAFDAC. (2019). Circulation of falsified antimalarials and antibiotics in sub-Saharan Africa. *Pharmacovigilance/Post Marketing Surveillance Newsletter*, *12*(2). https://www.nafdac.gov.ng/wp-content/uploads/Files/Resources/Pharmacovigilance_Newsletter/2019-Vol-12-No-2-Circulation-of-falsified-antimalaria-and-antibiotics-in-subsaharan-Africa.pdf

[CIT0015] NAFDAC. (2020). *Public Alert No. 012/2020 – Presence of suspected falsified SA’A TRIM (sulfamethoxazole) circulating in an illicit market in Chad*. https://nafdac.gov.ng/public-alert-no-012-2020-presence-of-suspected-falsified-saa-trim-sulfamethoxazole-circulating-in-an-illicit-market-in-chad/

[CIT0016] NAFDAC. (n.d.). *NAFDAC Greenbook – Nigeria’s registered drug product database.* National Agency for Food and Drug Administration and Control*.* Retrieved August 2, 2024, from https://greenbook.nafdac.gov.ng/

[CIT0017] Pan, H., & Ba-Thein, W. (2018). Diagnostic accuracy of Global Pharma Health Fund Minilab in assessing pharmacopoeial quality of antimicrobials. *The American Journal of Tropical Medicine and Hygiene*, *98*(1), 344–348. 10.4269/ajtmh.17-0289 29141717 PMC5928700

[CIT0018] Risha, P., Msuya, Z., Ndomondo-Sigonda, M., & Layloff, T. (2006). Proficiency testing as a tool to assess the performance of visual TLC quantitation estimates. *Journal of AOAC International*, *89*(5), 1300–1304. 10.1093/jaoac/89.5.1300 17042179

[CIT0019] Schäfermann, S., Hauk, C., Wemakor, E., Neci, R., Mutombo, G., Ngah Ndze, E., Cletus, T., Nyaah, F., Pattinora, M., Wistuba, D., Helmle, I., Häfele-Abah, C., Gross, H., & Heide, L. (2020). Substandard and falsified antibiotics and medicines against noncommunicable diseases in western Cameroon and northeastern Democratic Republic of Congo. *The American Journal of Tropical Medicine and Hygiene*, *103*(2), 894–908. 10.4269/ajtmh.20-0184 32394884 PMC7410427

[CIT0020] Schiavetti, B., Wynendaele, E., Melotte, V., Van der Elst, J., De Spiegeleer, B., & Ravinetto, R. (2020). A simplified checklist for the visual inspection of finished pharmaceutical products: A way to empower frontline health workers in the fight against poor-quality medicines. *Journal of Pharmaceutical Policy and Practice*, *13*(1), 9. 10.1186/s40545-020-00211-9 32377348 PMC7193355

[CIT0021] USP. (2020). *USP technology review: Global Pharma Health Fund (GPHF) – Minilab™*. Technology Review Program. https://www.usp.org/sites/default/files/usp/document/our-work/global-public-health/2020-usp-technology-review-global-pharma-health-fund-minilab.pdf

[CIT0022] Waffo Tchounga, C. A., Sacré, P.-Y., Ravinetto, R., Lieberman, M., Hamuli Ciza, P., Ngono Mballa, R., Ziemons, E., Hubert, P., & Djang’Eing’A Marini, R. (2023). Usefulness of medicine screening tools in the frame of pharmaceutical post-marketing surveillance. *PLoS One*, *18*(8), e0289865. 10.1371/journal.pone.0289865 37566594 PMC10420354

[CIT0023] WHO. (2011). *Survey of the quality of selected antimalarial medicines circulating in six countries of sub-Saharan Africa.* https://www.afro.who.int/sites/default/files/2017-06/WHO_QAMSA_report.pdf

[CIT0024] WHO. (2017a). *A study on the public health and socioeconomic impact of substandard and falsified medical products.* https://www.who.int/publications/i/item/9789241513432

[CIT0025] WHO. (2017b). *WHO global surveillance and monitoring system for substandard and falsified medical products.* https://apps.who.int/iris/handle/10665/326708

[CIT0026] WHPA. (2011). *Toolkit – Be aware, take action. Tool for visual inspection of medicines.* https://www.whpa.org/news-resources/toolkit-be-aware-take-action

[CIT0027] Zambrzycki, S. C., Caillet, C., Vickers, S., Bouza, M., Donndelinger, D. V., Geben, L. C., Bernier, M. C., Newton, P. N., & Fernández, F. M. (2021). Laboratory evaluation of twelve portable devices for medicine quality screening. *PLOS Neglected Tropical Diseases*, *15*(9), e0009360. 10.1371/journal.pntd.0009360 34591844 PMC8483346

[CIT0028] ZLF. (n.d.). *ZLF pharmaceutical limited.* http://www.zlfpharma.com/

