# Supplementary material for: Screening for substandard and falsified medicines in Nigeria using visual inspection and GPHF-Minilab analysis: lessons learnt for future training of health workers and pharmacy personnel
Source: J Pharm Policy Pract. 2024 Dec 9;17(1):2432471. doi: 10.1080/20523211.2024.2432471 (PMC11632929; doi:10.1080/20523211.2024.2432471)
Supplement: Supplementary_Table_S3_Visual_inspection_checklist_Revised.pdf [file JPPP_A_2432471_SM8527.pdf]

Supplementary Table S3. Checklist for the visual inspection of medicines

The checklist presented in the following two pages was adapted from Schiavetti et al., J Pharm Policy Pract 13:9 (2020), introducing the following modifications:

- A field to record manufacturer and batch number was added, to ensure traceability of the visual inspection results to the respective sample.
- In addition to the replies "yes" or "no", a further option "not applicable" was included.
- Some questions were reworded so that the reply "no" always signifies a visual deficiency.
- Some questions were reworded for simplicity and clarity.
- Section (B) was renamed to "Labeling" rather than "Identification" for clarity.
- The terms "external" and "internal" packaging were renamed to "secondary" and "primary" packaging. In the field test of this checklist, confusion of the local pharmacy staff about the meaning of "external" and "internal" packaging had been observed. Also, in the case of bulk containers there is only a primary packaging which, however, is not well described by the term "internal packaging".
- Some questions were added due to observations of visual deficiencies among substandard and falsified medicines in the present study:
    - No. 10 and 17: legibility of batch number and expiry date
    - No. 11 and 18: spelling of the active pharmaceutical ingredient
    - No. 20 and 21: consistency of batch numbers and expiry dates given on the secondary and on the primary packaging.
    - No. 31: complete filling of blister compartments
    - No. 32: presence of an unpleasant odor
- A section with specific questions for samples collected in Nigeria was added (= section B.3), concerning the NAFDAC registration numbers as well as PIN codes of the NAFDAC Mobile Authentication Scheme. These questions may have to be adapted or omitted for use in other countries.
- Questions no. 20 and 24 of the original checklist were removed, since they were found not to be sufficiently clear to the pharmacy staff, or not sufficiently relevant for the identification of substandard or falsified medicines.

So far, this visual inspection checklist has only been tested for solid oral formulations and for powders for injection, not for liquid formulations or syrups.

VISUAL INSPECTION CHECKLIST

Name of the product: ____

Manufacturer: ____

Batch No: ______________________________

Date of inspection: _____/_____/____________

A. PACKAGING	Yes	Not applicable	No	Observations
1. Is there a primary[1] packaging?				
2. Is there a secondary[2] packaging?				
3. Is the packaging intact?				
4. Does the packaging provide information on the storage conditions of the medicine?				
**B. LABELING**				
B.1 Does the ***secondary*** *packaging state the following information?*				
5. Name of the active ingredient or in case of a combination product the name of all active ingredients				
6. Amount of active ingredient				
7. Name and address of the manufacturer and/or the name and address of the marketing authorization holder[3]				
8. Batch number				
9. Expiry date				
10. Are the stated batch number and expiry date legible?				
11. Is the name of the active ingredient spelled correctly?				
B.2 Does the ***primary*** *packaging state the following information?*				
12. Name of the active ingredient				
13. Amount of active ingredient				
14. Name and address of the manufacturer and/or the name and address of the marketing authorization holder				
15. Batch number				
16. Expiry date				
17. Are the stated batch number and expiry date legible?				
18. Is the name of the active ingredient spelled correctly?				
19. Is the stated amount of the active ingredient the same on the secondary and primary packaging, and on every blister/vial?				
20. Is the batch number the same on the secondary and primary packaging, and on every blister/vial?				
21. Is the expiry date the same on the secondary and primary packaging, and on every blister/vial?				
B.3 Specific questions for samples collected in Nigeria				
22. Does the packaging carry a NAFDAC Registration Number?				
23. If the answer to 22 is yes, can the NAFDAC Registration Number be verified in the NAFDAC Greenbook? (https://greenbook.nafdac.gov.ng/)				

Adapted by Lächele et al. from B. Schiavetti et al. (J Pharm Policy Pract 13:9 (2020).

VISUAL INSPECTION CHECKLIST

24. If the answer to 22 or 23 is no, is the manufacturer listed in the NAFDAC Greenbook? (https://greenbook.nafdac.gov.ng/manufacturers)				
25. If the answer to 24 is no, can the manufacturer be found by a Google search on the internet?				
26. In case of anti-infectives: does the packaging carry a PIN for the Mobile Authentication Service (MAS) scheme?				
27. If the answer to 26 is yes, is the opaque covering of the MAS PIN intact (= the PIN has not been scratched free yet)?				
28. In case you decide to scratch the PIN free and text it by SMS to the given phone number: does the response correctly confirm the authenticity of the product?				
**C. PHYSICAL APPEARANCE**				
C.1 Tablets or capsules				
29. Have all tablets/capsules the same shape, dimension, colour, marks/embossings?				
30. Are the tablets/capsules free from cracks, erosion, stains, foreign particles?				
31. If packaged in blisters: Are all blister compartments filled with one tablet each (= no compartment is empty)?				
32. If packaged in bulk: Are the tablets/capsules free from an unpleasant odour?				
C.2 Powders for suspension and syrups				
33. Is the powder/syrup homogeneous, free from lumps, clots, foreign particles?				
34. Is the color of the powder/syrup homogeneous?				
35. Is a dosing device provided with the product?				
36. Are there clear instructions for preparing the oral liquid solution (type and quantity of liquid to be used, and how)?[4]				
37. Is there a mark on the bottle indicating the final volume of the re-suspended powder?[4]				
C.3 Sterile liquids, powders for injection				
38. Is the closure of the primary packaging intact and airtight?				
39. Is the liquid/powder homogeneous, free from lumps/clots, foreign particles?				
40. Is the color of the liquid/powder homogeneous?				

[1] Primary packaging is the packaging which is in direct contact with the medicine e.g. a blister strips or a vial. (If tablets inside a bulk container are contained in a simple plastic bag which does not carry any printed information, write "plastic bag" in Observations, and tick "Not applicable" in questions 12 to 21).

[2] Secondary packaging is the packaging that envelops the primary packaging, e.g. a cardboard box containing blister strips with tablets or different vials.

[3] Manufacturer is the company which manufactured the product. Marketing Authorization Holder is the company which has a license to market the product in a specific country; it may be identical to the manufacturer, or be a different company.

[4] Answer this question only if the formulation is a powder that should be resuspended.
