# Supplementary material for: Screening for substandard and falsified medicines in Nigeria using visual inspection and GPHF-Minilab analysis: lessons learnt for future training of health workers and pharmacy personnel
Source: J Pharm Policy Pract. 2024 Dec 9;17(1):2432471. doi: 10.1080/20523211.2024.2432471 (PMC11632929; doi:10.1080/20523211.2024.2432471)
Supplement: Supplementary_Figures_S1_to_S21_revised.pptx [file JPPP_A_2432471_SM8526.pptx]

## Slide 1
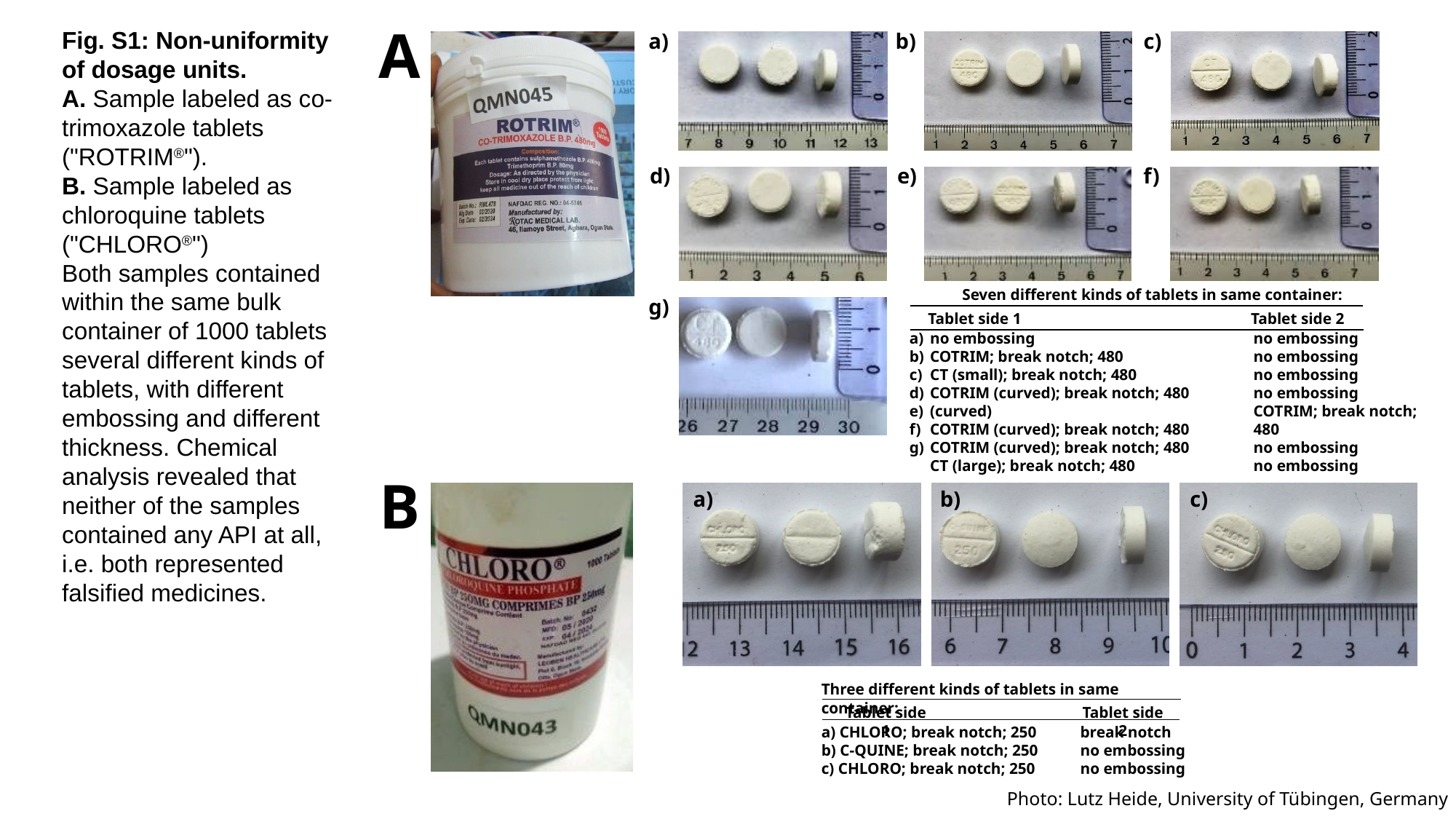

A
Fig. S1: Non-uniformity of dosage units.
A. Sample labeled as co-trimoxazole tablets ("ROTRIM®").
B. Sample labeled as chloroquine tablets ("CHLORO®")
Both samples contained within the same bulk container of 1000 tablets several different kinds of tablets, with different embossing and different thickness. Chemical analysis revealed that neither of the samples contained any API at all, i.e. both represented falsified medicines.
a)
b)
c)
d)
e)
f)
Seven different kinds of tablets in same container:
g)
Tablet side 1
Tablet side 2
a)
b)
c)
d)
e)
f)
g)
no embossing
COTRIM; break notch; 480
CT (small); break notch; 480
COTRIM (curved); break notch; 480 (curved)
COTRIM (curved); break notch; 480
COTRIM (curved); break notch; 480
CT (large); break notch; 480
no embossing
no embossing
no embossing
no embossing
COTRIM; break notch; 480
no embossing
no embossing
B
b)
a)
c)
Three different kinds of tablets in same container:
Tablet side 1
Tablet side 2
a) CHLORO; break notch; 250
b) C-QUINE; break notch; 250
c) CHLORO; break notch; 250
break notch
no embossing
no embossing
Photo: Lutz Heide, University of Tübingen, Germany

## Slide 2
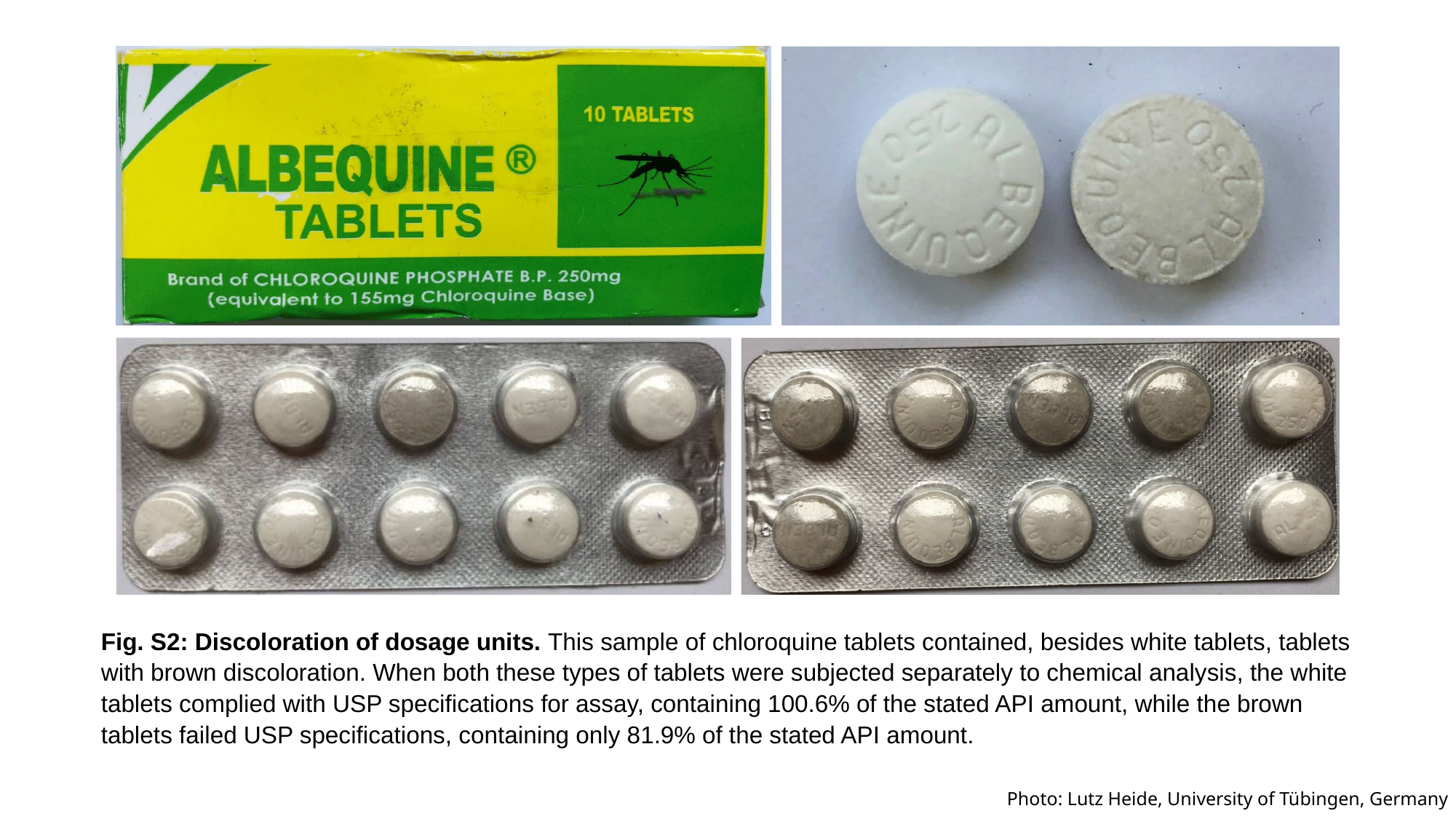

Fig. S2: Discoloration of dosage units. This sample of chloroquine tablets contained, besides white tablets, tablets with brown discoloration. When both these types of tablets were subjected separately to chemical analysis, the white tablets complied with USP specifications for assay, containing 100.6% of the stated API amount, while the brown tablets failed USP specifications, containing only 81.9% of the stated API amount.
Photo: Lutz Heide, University of Tübingen, Germany

## Slide 3
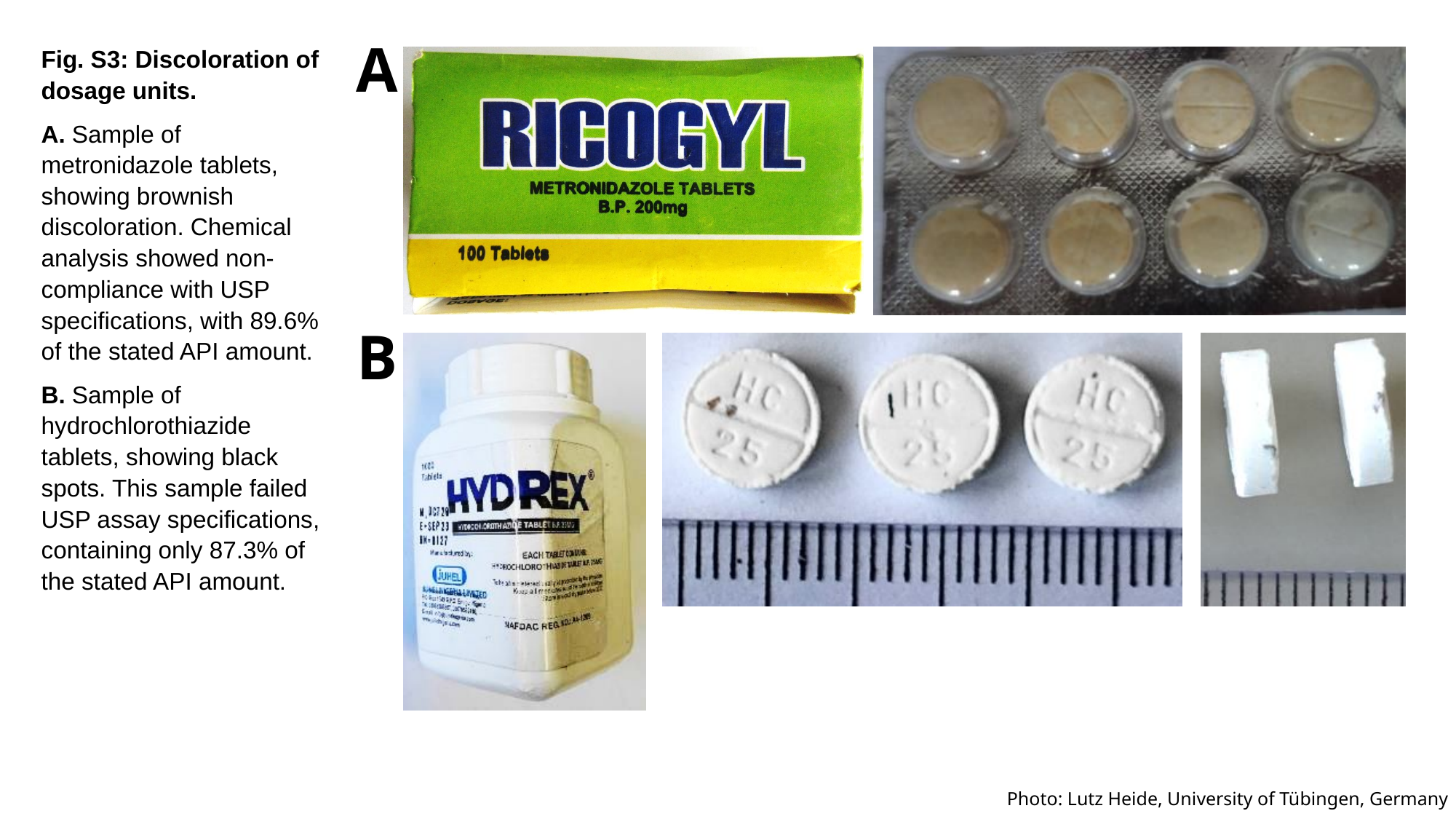

A
Fig. S3: Discoloration of dosage units.
A. Sample of metronidazole tablets, showing brownish discoloration. Chemical analysis showed non-compliance with USP specifications, with 89.6% of the stated API amount.
B. Sample of hydrochlorothiazide tablets, showing black spots. This sample failed USP assay specifications, containing only 87.3% of the stated API amount.
B
Photo: Lutz Heide, University of Tübingen, Germany

## Slide 4
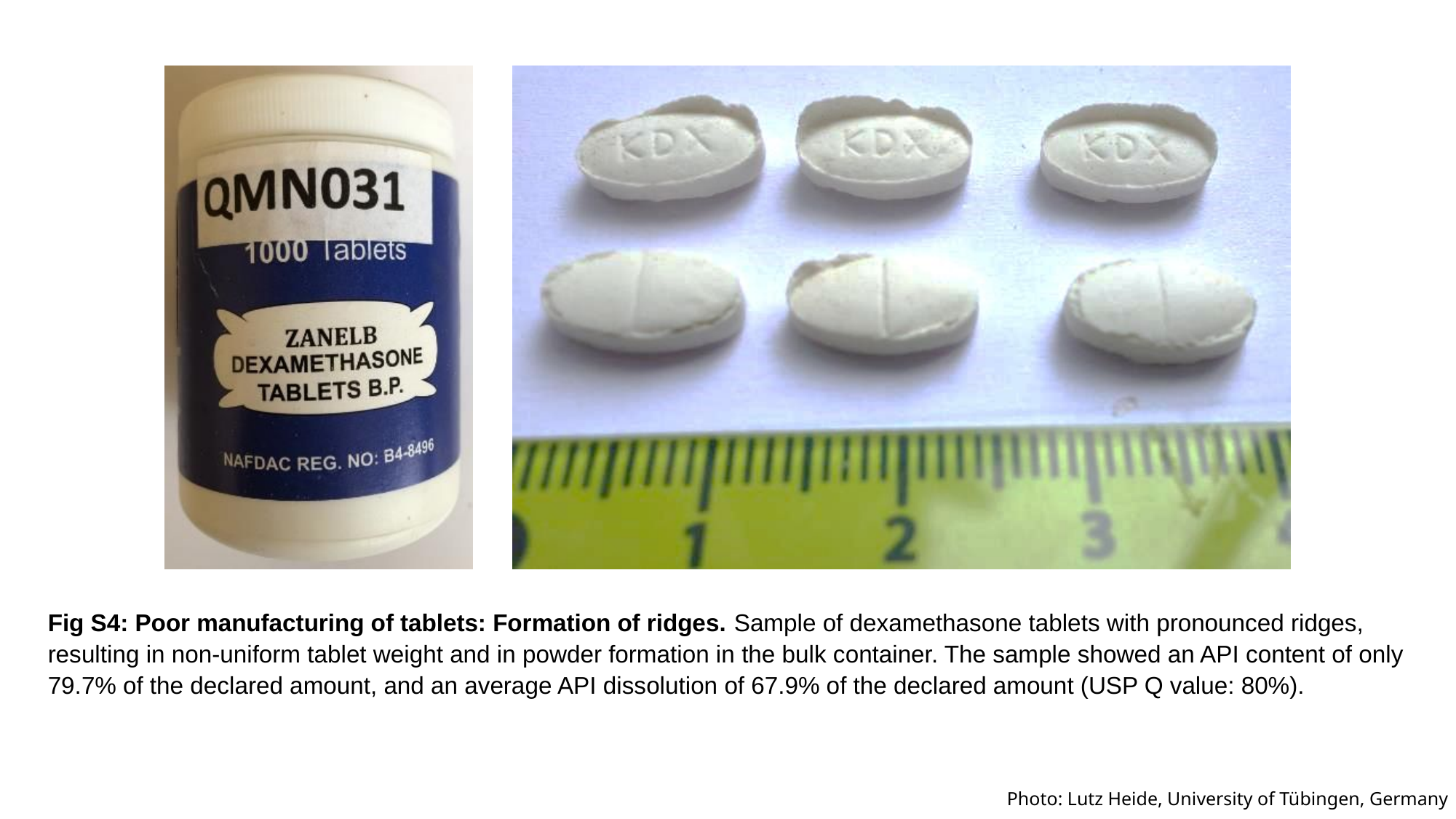

Fig S4: Poor manufacturing of tablets: Formation of ridges. Sample of dexamethasone tablets with pronounced ridges, resulting in non-uniform tablet weight and in powder formation in the bulk container. The sample showed an API content of only 79.7% of the declared amount, and an average API dissolution of 67.9% of the declared amount (USP Q value: 80%).
Photo: Lutz Heide, University of Tübingen, Germany

## Slide 5
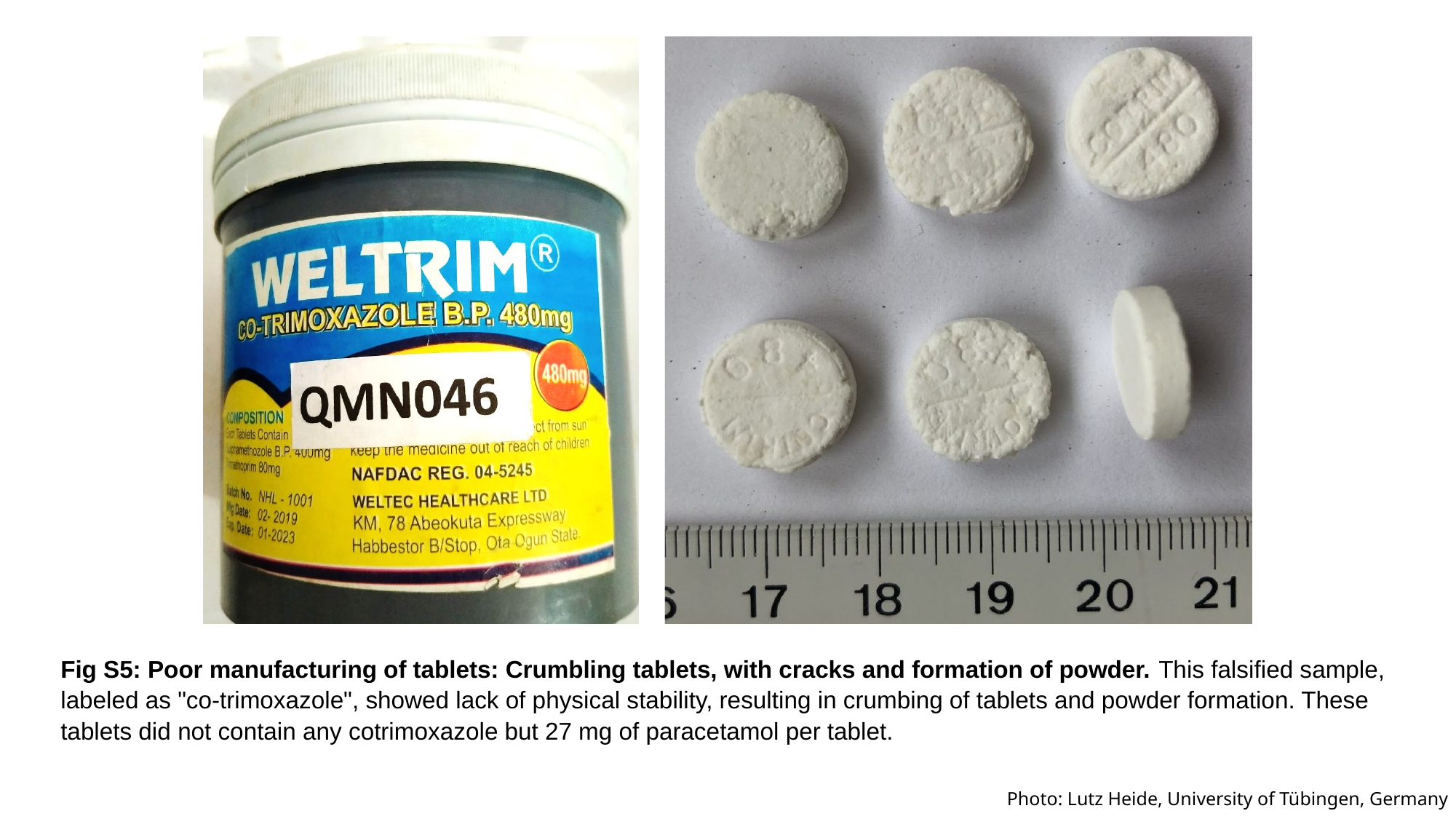

Fig S5: Poor manufacturing of tablets: Crumbling tablets, with cracks and formation of powder. This falsified sample, labeled as "co-trimoxazole", showed lack of physical stability, resulting in crumbing of tablets and powder formation. These tablets did not contain any cotrimoxazole but 27 mg of paracetamol per tablet.
Photo: Lutz Heide, University of Tübingen, Germany

## Slide 6
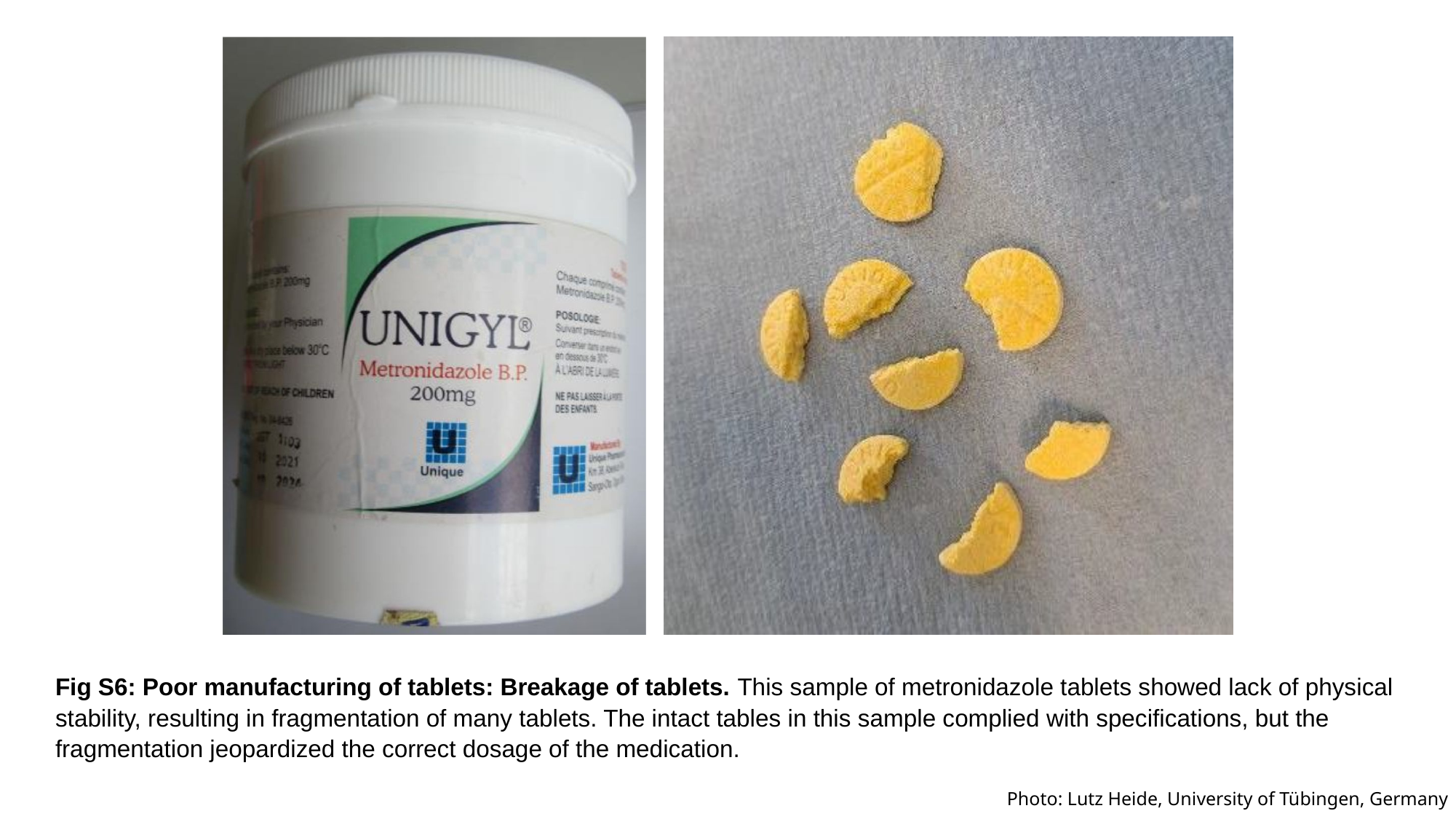

Fig S6: Poor manufacturing of tablets: Breakage of tablets. This sample of metronidazole tablets showed lack of physical stability, resulting in fragmentation of many tablets. The intact tables in this sample complied with specifications, but the fragmentation jeopardized the correct dosage of the medication.
Photo: Lutz Heide, University of Tübingen, Germany

## Slide 7
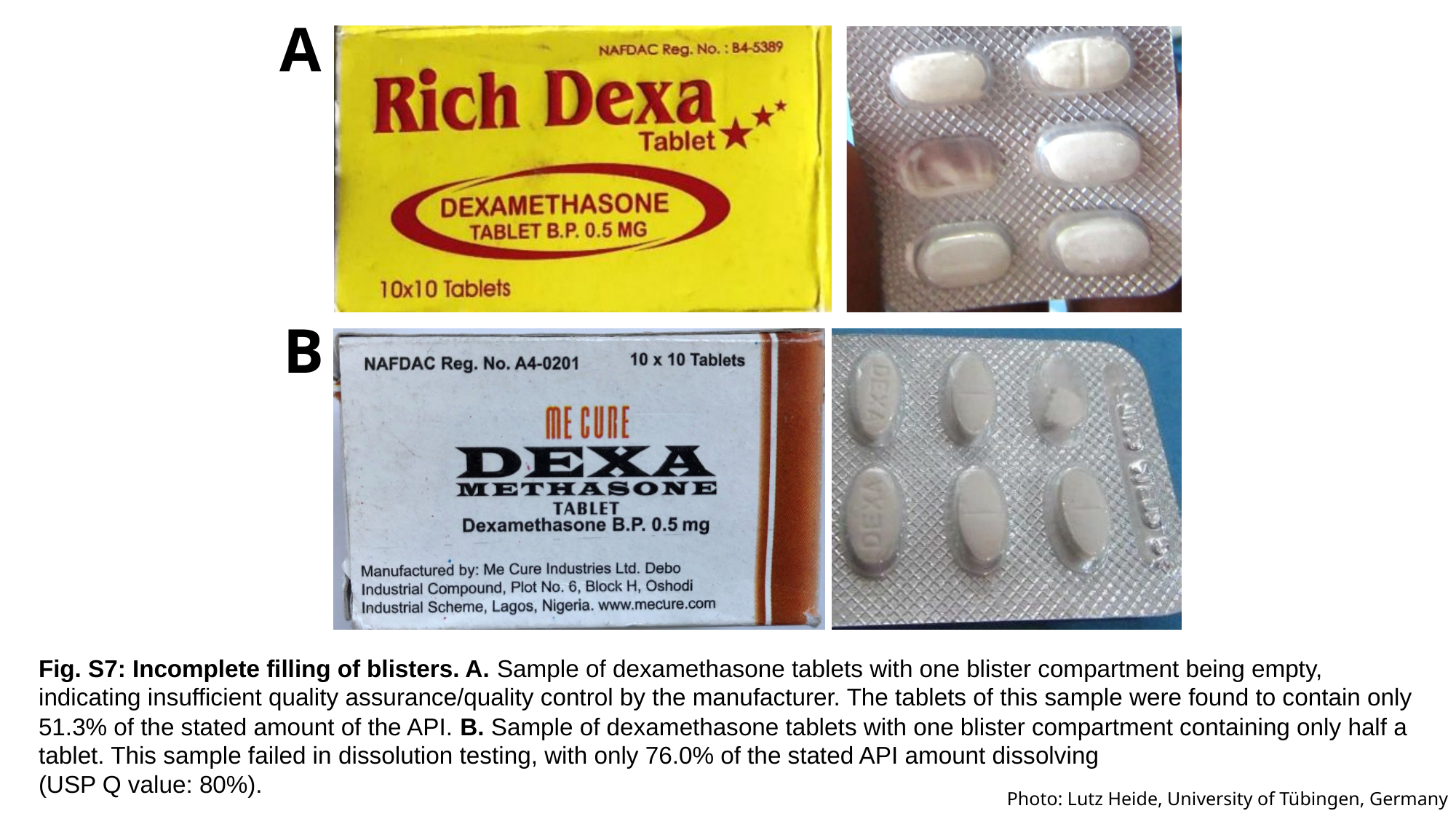

A
B
Fig. S7: Incomplete filling of blisters. A. Sample of dexamethasone tablets with one blister compartment being empty, indicating insufficient quality assurance/quality control by the manufacturer. The tablets of this sample were found to contain only 51.3% of the stated amount of the API. B. Sample of dexamethasone tablets with one blister compartment containing only half a tablet. This sample failed in dissolution testing, with only 76.0% of the stated API amount dissolving
(USP Q value: 80%).
Photo: Lutz Heide, University of Tübingen, Germany

## Slide 8
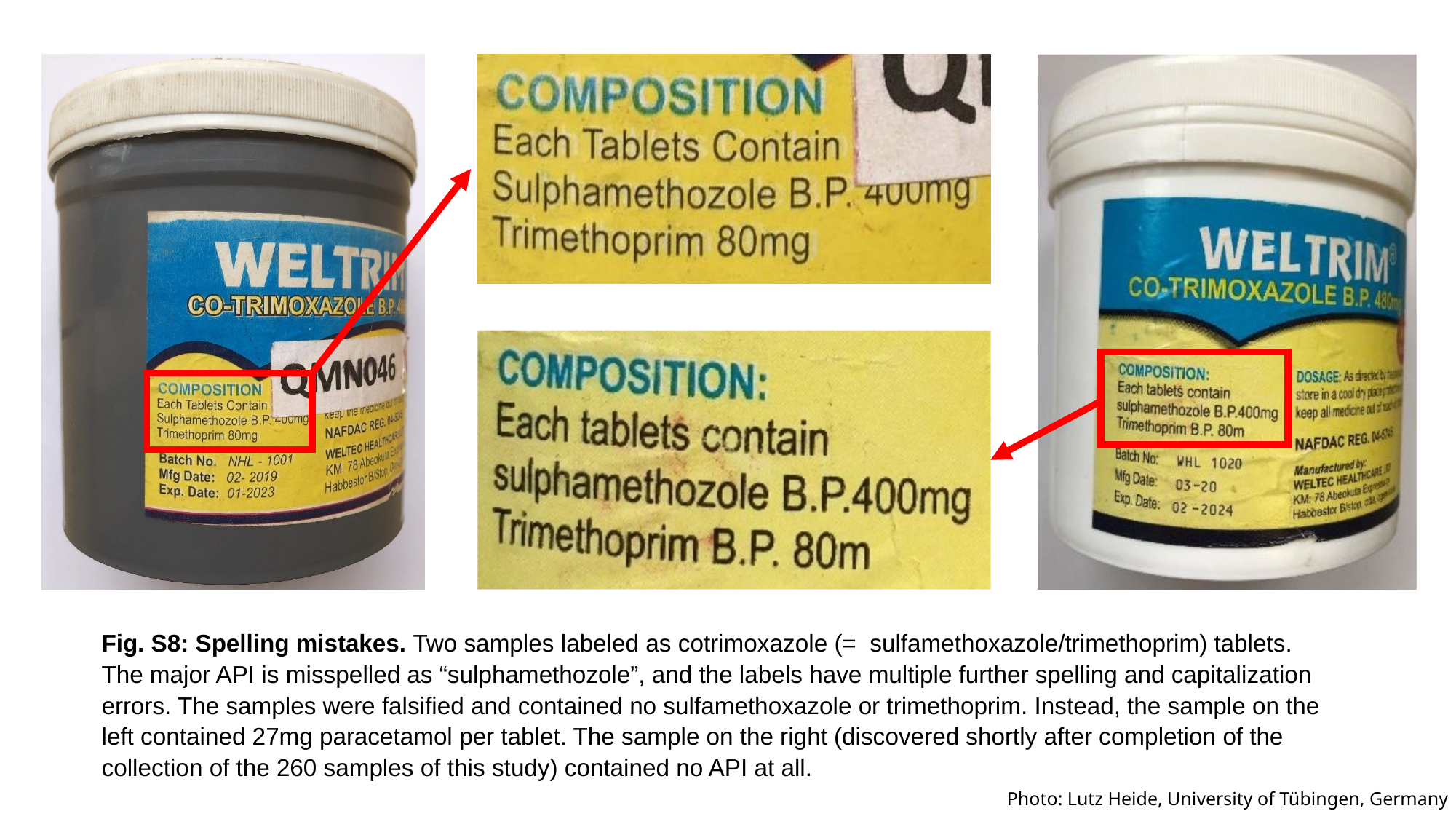

Fig. S8: Spelling mistakes. Two samples labeled as cotrimoxazole (= sulfamethoxazole/trimethoprim) tablets. The major API is misspelled as “sulphamethozole”, and the labels have multiple further spelling and capitalization errors. The samples were falsified and contained no sulfamethoxazole or trimethoprim. Instead, the sample on the left contained 27mg paracetamol per tablet. The sample on the right (discovered shortly after completion of the collection of the 260 samples of this study) contained no API at all.
Photo: Lutz Heide, University of Tübingen, Germany

## Slide 9
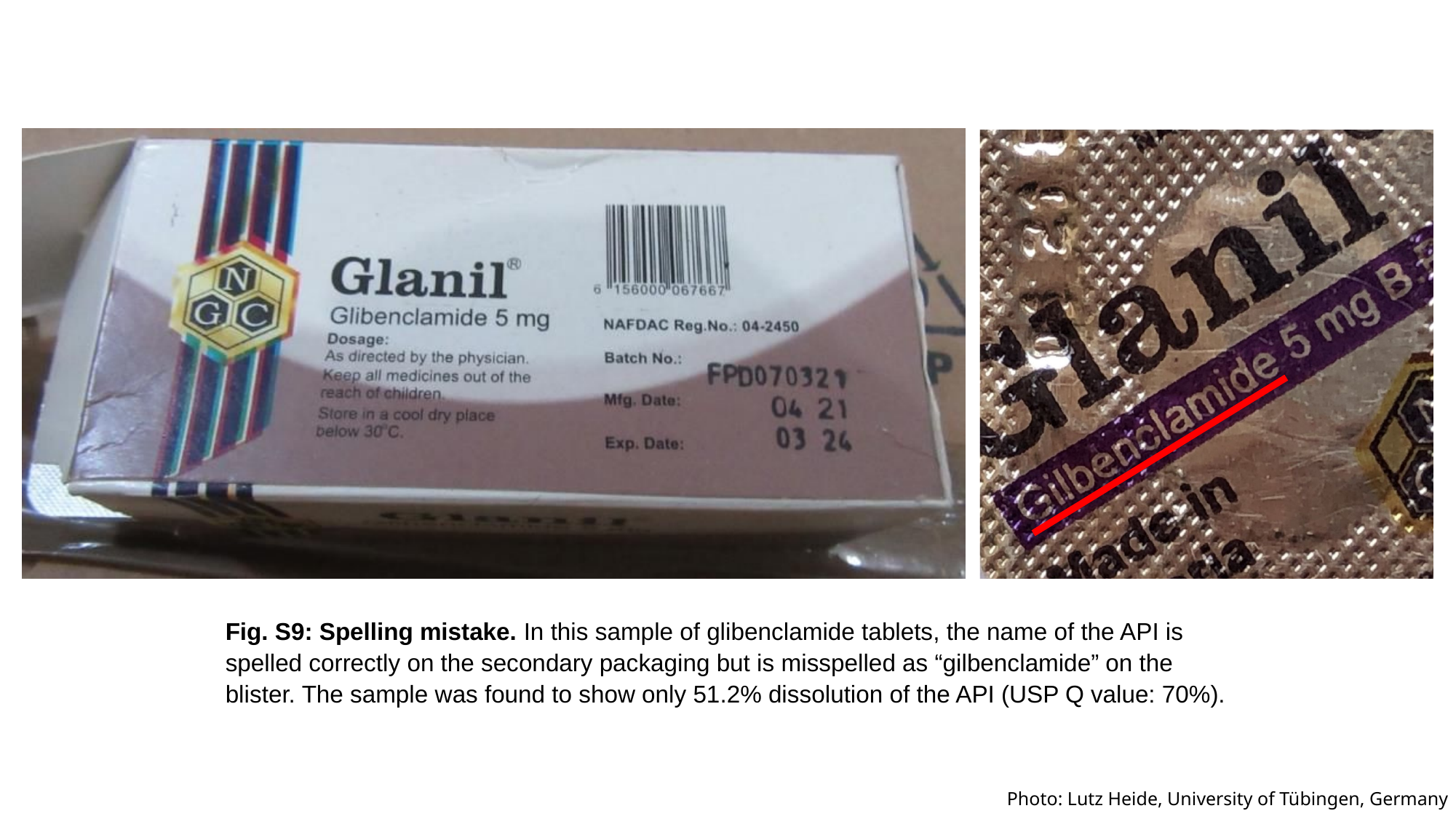

Fig. S9: Spelling mistake. In this sample of glibenclamide tablets, the name of the API is spelled correctly on the secondary packaging but is misspelled as “gilbenclamide” on the blister. The sample was found to show only 51.2% dissolution of the API (USP Q value: 70%).
Photo: Lutz Heide, University of Tübingen, Germany

## Slide 10
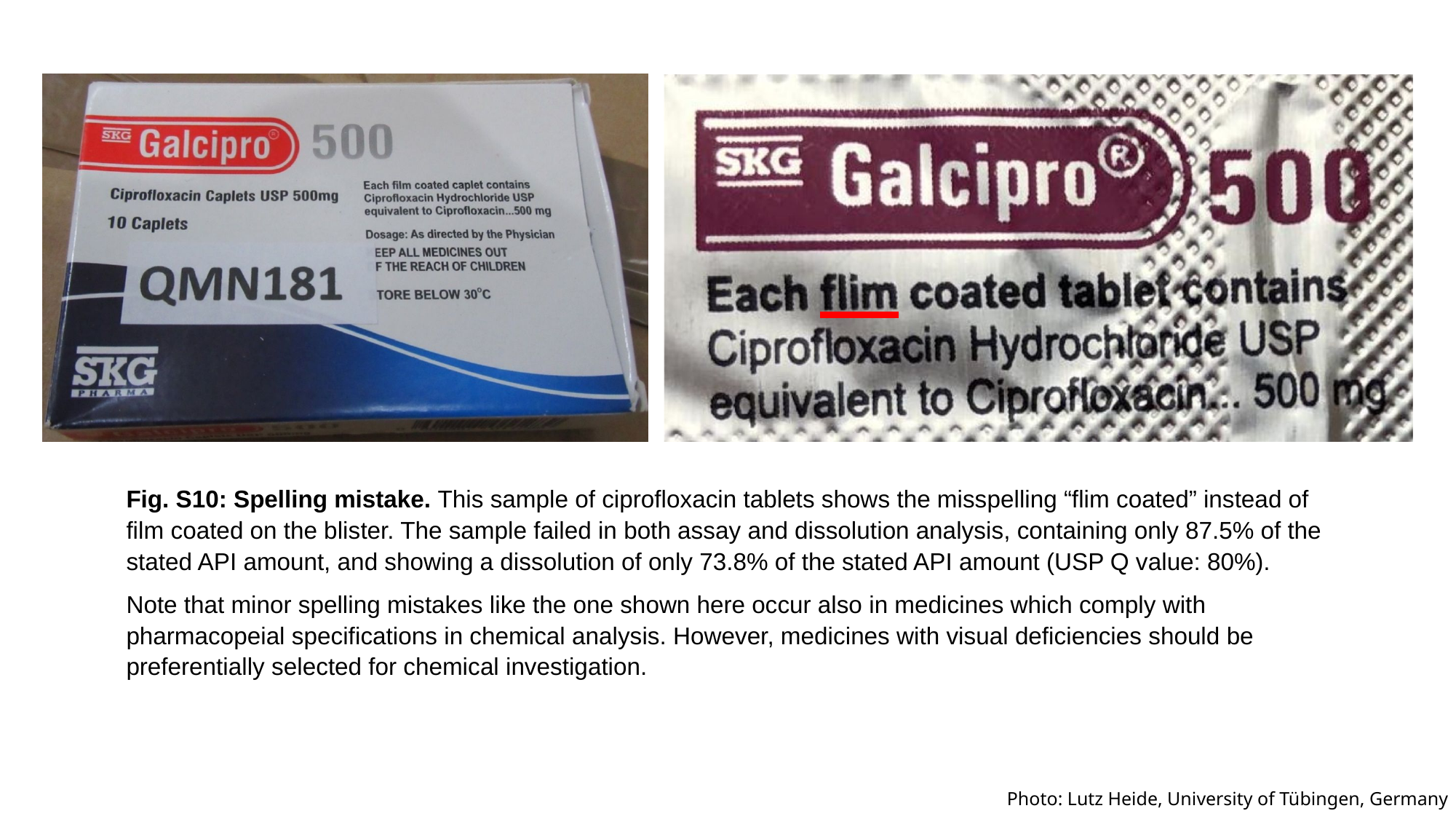

Fig. S10: Spelling mistake. This sample of ciprofloxacin tablets shows the misspelling “flim coated” instead of film coated on the blister. The sample failed in both assay and dissolution analysis, containing only 87.5% of the stated API amount, and showing a dissolution of only 73.8% of the stated API amount (USP Q value: 80%).
Note that minor spelling mistakes like the one shown here occur also in medicines which comply with pharmacopeial specifications in chemical analysis. However, medicines with visual deficiencies should be preferentially selected for chemical investigation.
Photo: Lutz Heide, University of Tübingen, Germany

## Slide 11
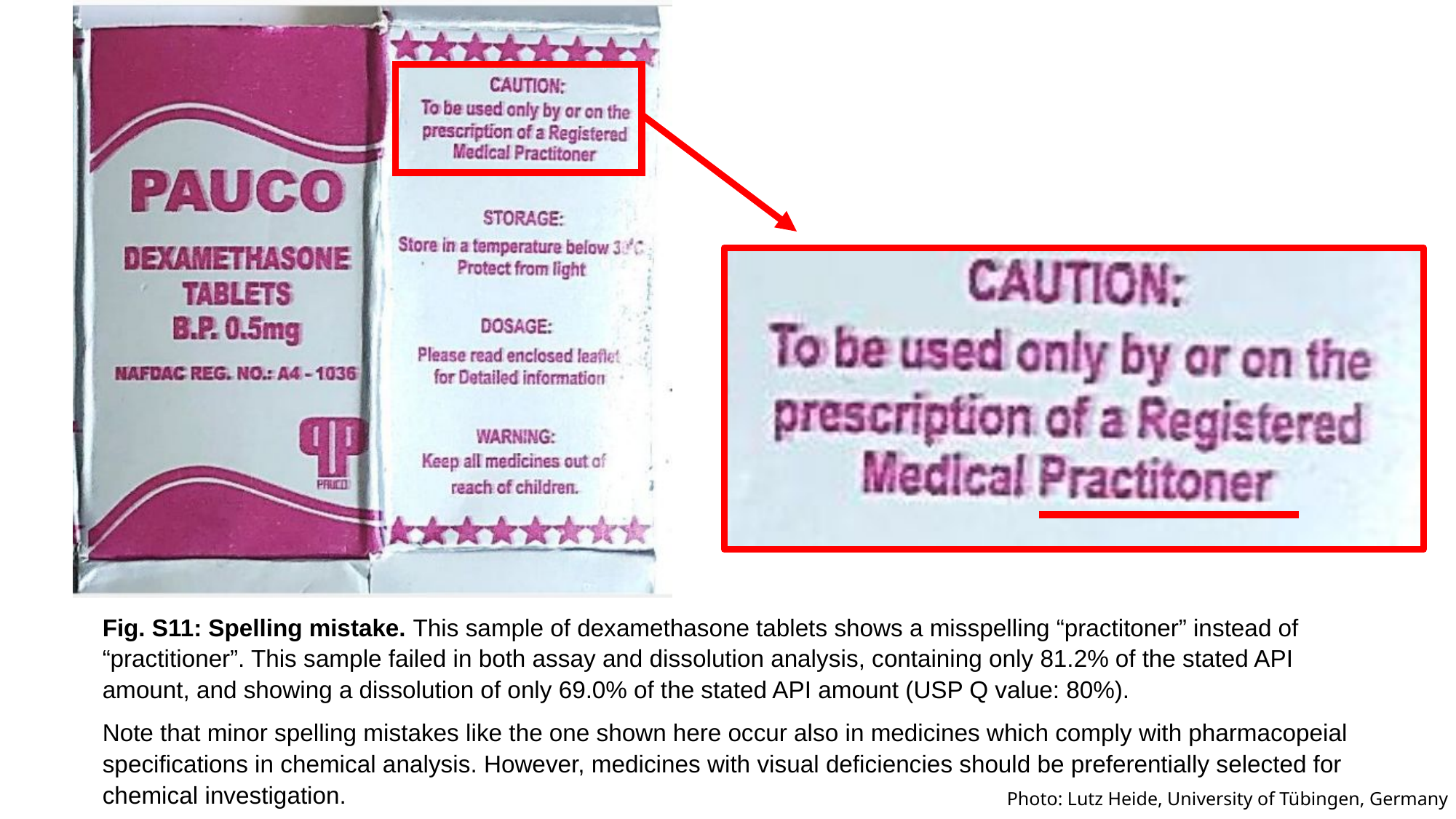

Fig. S11: Spelling mistake. This sample of dexamethasone tablets shows a misspelling “practitoner” instead of “practitioner”. This sample failed in both assay and dissolution analysis, containing only 81.2% of the stated API amount, and showing a dissolution of only 69.0% of the stated API amount (USP Q value: 80%).
Note that minor spelling mistakes like the one shown here occur also in medicines which comply with pharmacopeial specifications in chemical analysis. However, medicines with visual deficiencies should be preferentially selected for chemical investigation.
Photo: Lutz Heide, University of Tübingen, Germany

## Slide 12
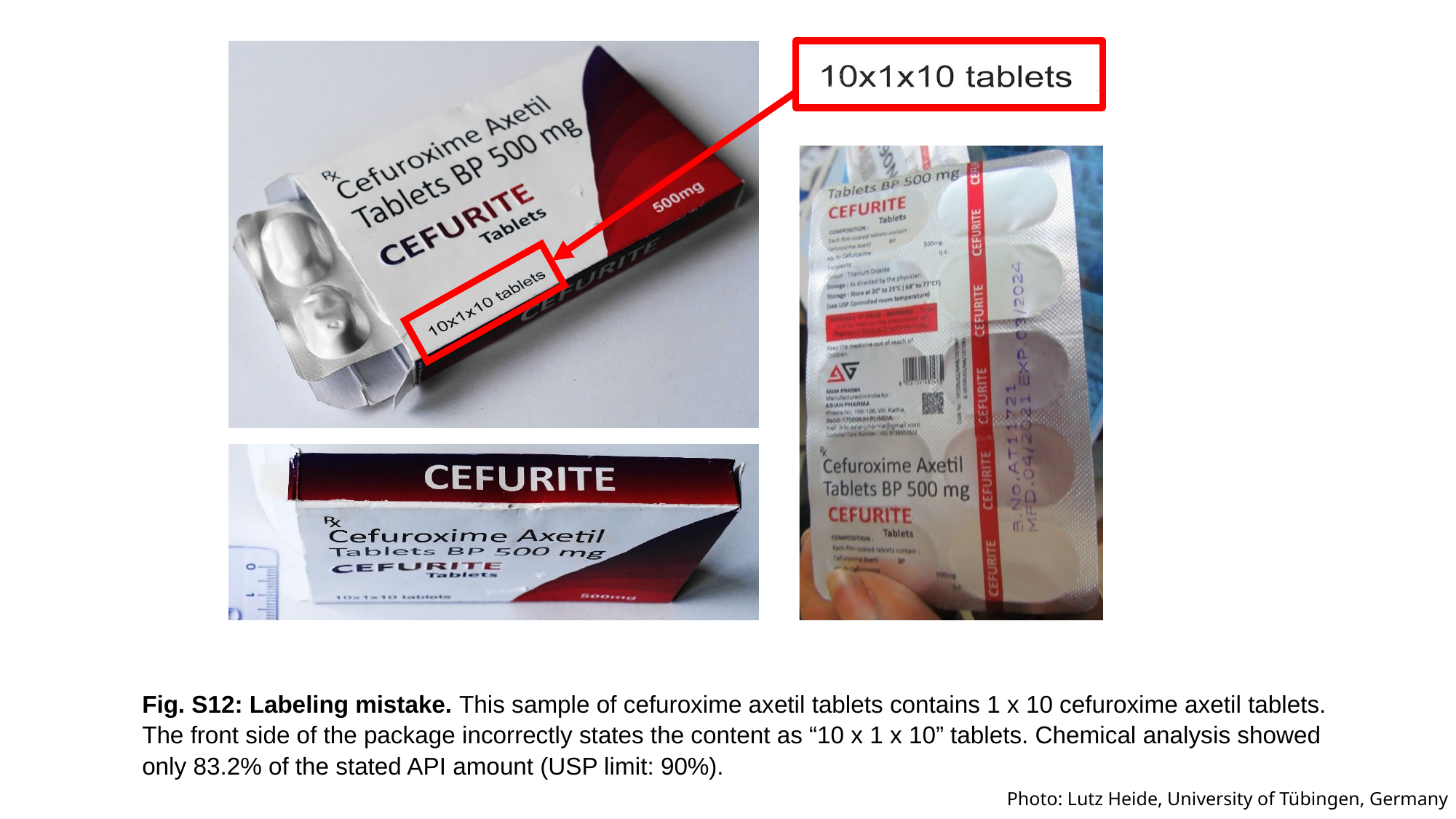

Fig. S12: Labeling mistake. This sample of cefuroxime axetil tablets contains 1 x 10 cefuroxime axetil tablets. The front side of the package incorrectly states the content as “10 x 1 x 10” tablets. Chemical analysis showed only 83.2% of the stated API amount (USP limit: 90%).
Photo: Lutz Heide, University of Tübingen, Germany

## Slide 13
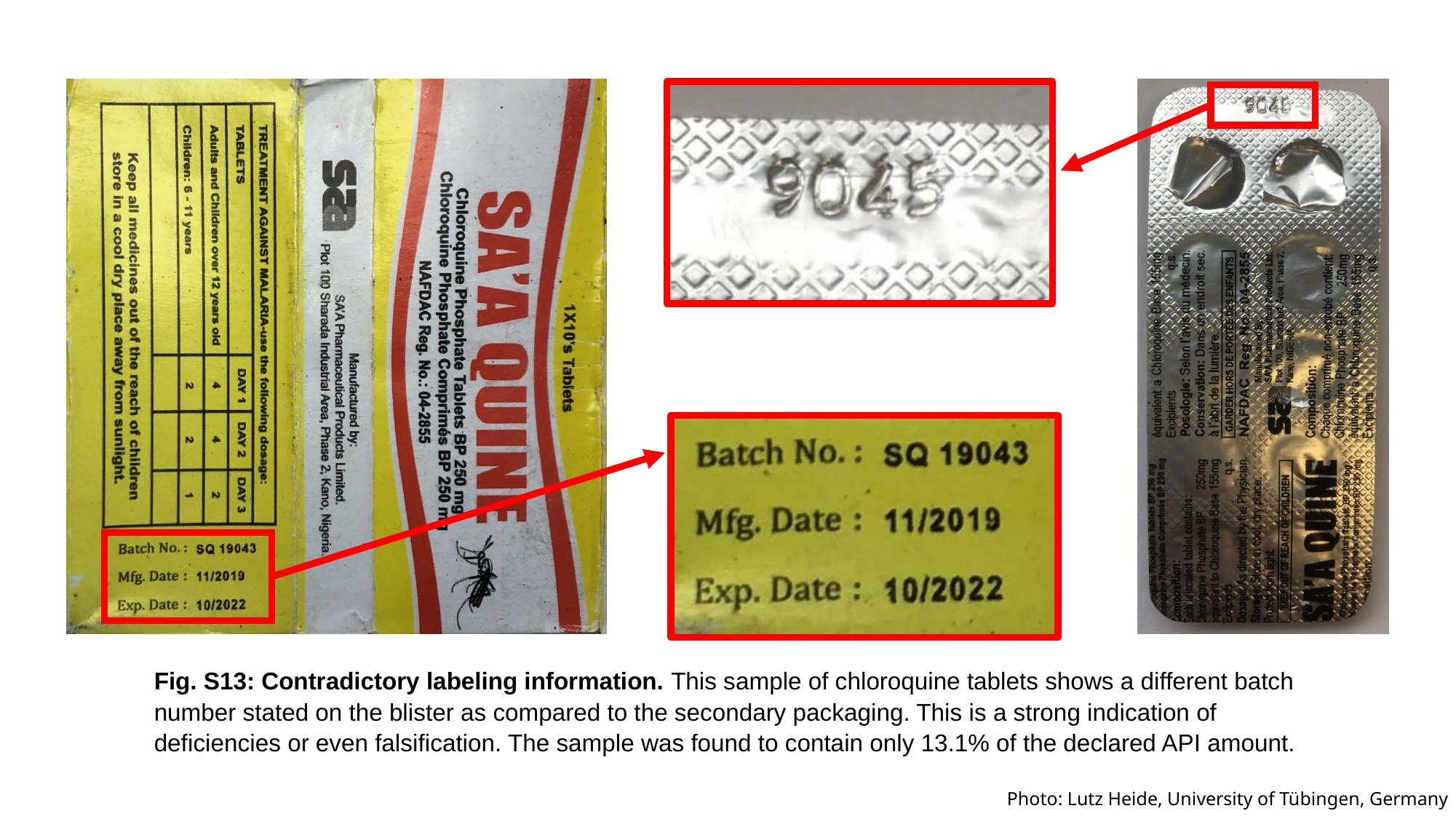

Fig. S13: Contradictory labeling information. This sample of chloroquine tablets shows a different batch number stated on the blister as compared to the secondary packaging. This is a strong indication of deficiencies or even falsification. The sample was found to contain only 13.1% of the declared API amount.
Photo: Lutz Heide, University of Tübingen, Germany

## Slide 14
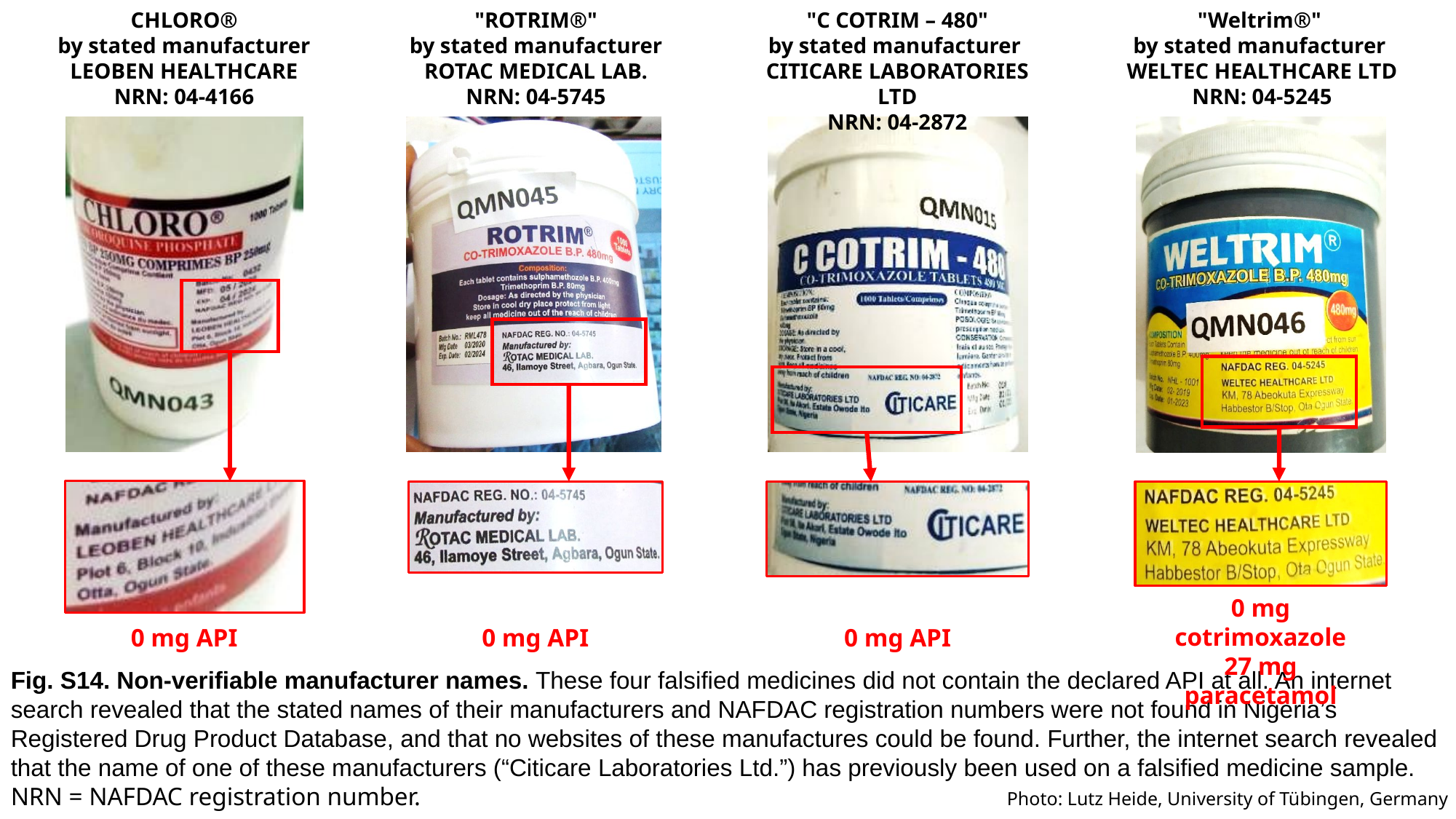

CHLORO®
by stated manufacturer LEOBEN HEALTHCARE
NRN: 04-4166
"ROTRIM®"
by stated manufacturer ROTAC MEDICAL LAB.
NRN: 04-5745
"C COTRIM – 480"
by stated manufacturer
CITICARE LABORATORIES LTD
NRN: 04-2872
"Weltrim®"
by stated manufacturer
WELTEC HEALTHCARE LTD
NRN: 04-5245
0 mg cotrimoxazole
27 mg paracetamol
0 mg API
0 mg API
0 mg API
Fig. S14. Non-verifiable manufacturer names. These four falsified medicines did not contain the declared API at all. An internet search revealed that the stated names of their manufacturers and NAFDAC registration numbers were not found in Nigeria’s Registered Drug Product Database, and that no websites of these manufactures could be found. Further, the internet search revealed that the name of one of these manufacturers (“Citicare Laboratories Ltd.”) has previously been used on a falsified medicine sample.
NRN = NAFDAC registration number.
Photo: Lutz Heide, University of Tübingen, Germany

## Slide 15
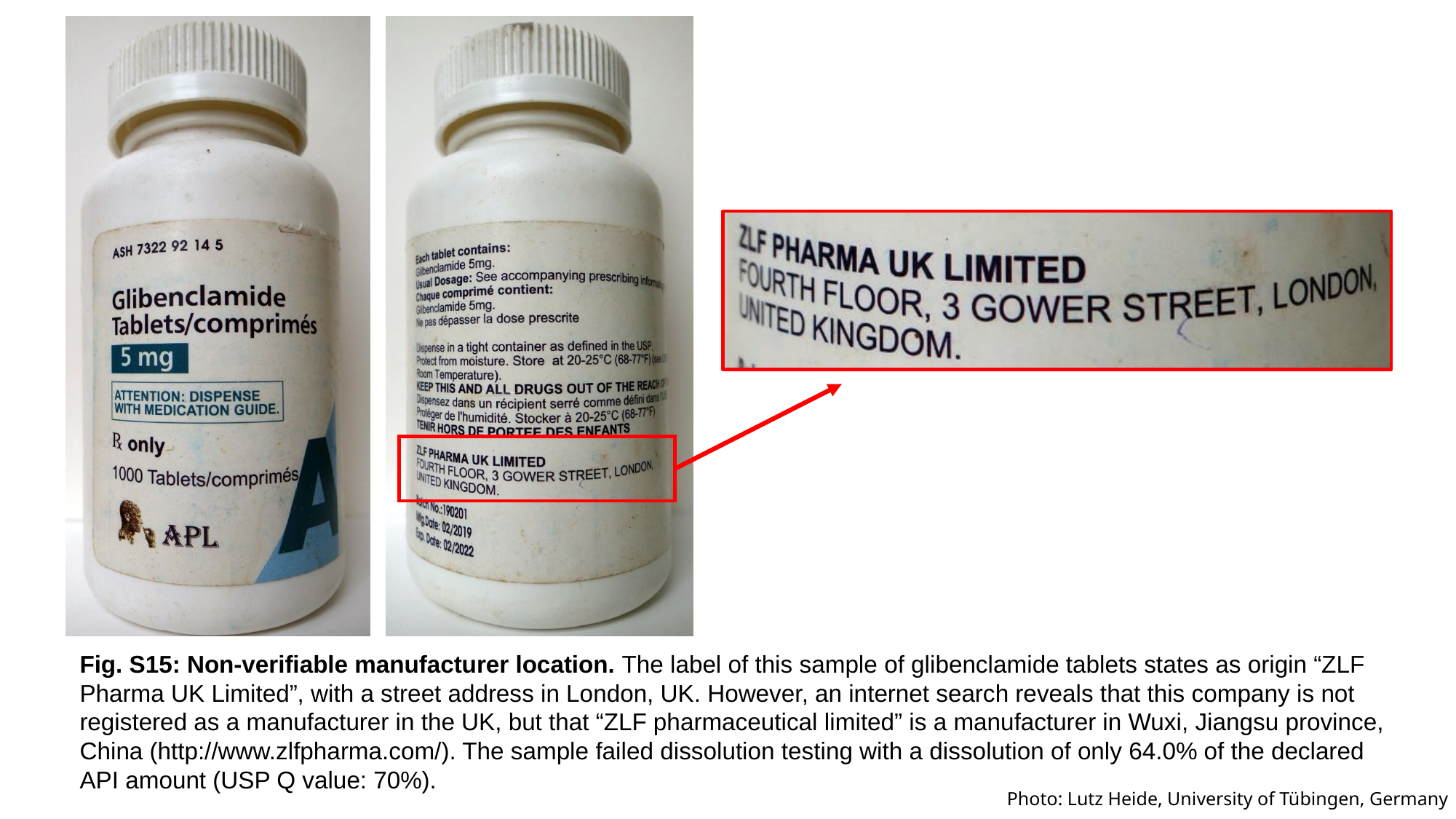

Fig. S15: Non-verifiable manufacturer location. The label of this sample of glibenclamide tablets states as origin “ZLF Pharma UK Limited”, with a street address in London, UK. However, an internet search reveals that this company is not registered as a manufacturer in the UK, but that “ZLF pharmaceutical limited” is a manufacturer in Wuxi, Jiangsu province, China (http://www.zlfpharma.com/). The sample failed dissolution testing with a dissolution of only 64.0% of the declared API amount (USP Q value: 70%).
Photo: Lutz Heide, University of Tübingen, Germany

## Slide 16
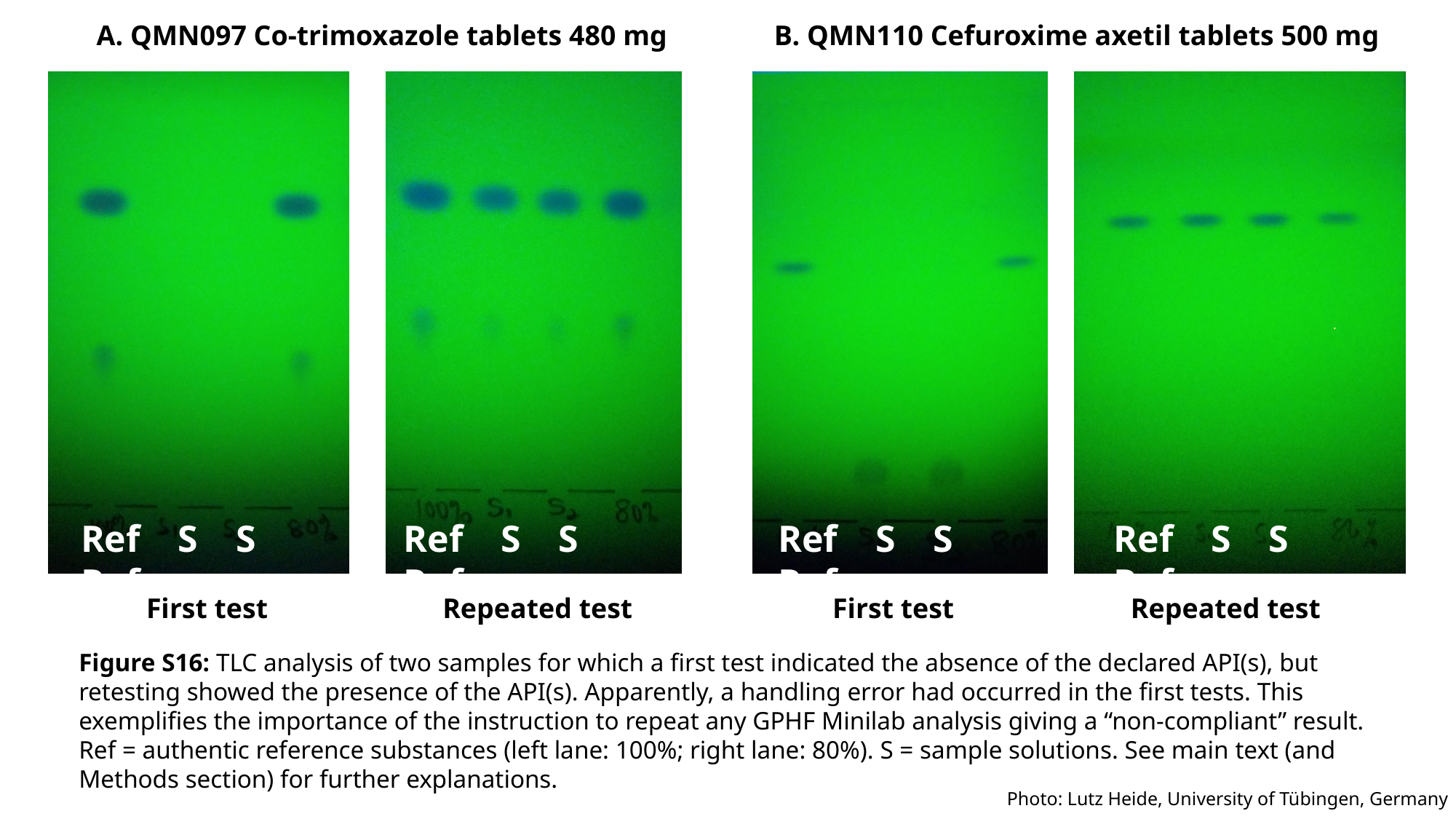

A. QMN097 Co-trimoxazole tablets 480 mg
B. QMN110 Cefuroxime axetil tablets 500 mg
Ref S S Ref
Ref S S Ref
Ref S S Ref
Ref S S Ref
First test
Repeated test
First test
Repeated test
Figure S16: TLC analysis of two samples for which a first test indicated the absence of the declared API(s), but retesting showed the presence of the API(s). Apparently, a handling error had occurred in the first tests. This exemplifies the importance of the instruction to repeat any GPHF Minilab analysis giving a “non-compliant” result.
Ref = authentic reference substances (left lane: 100%; right lane: 80%). S = sample solutions. See main text (and Methods section) for further explanations.
Photo: Lutz Heide, University of Tübingen, Germany

## Slide 17
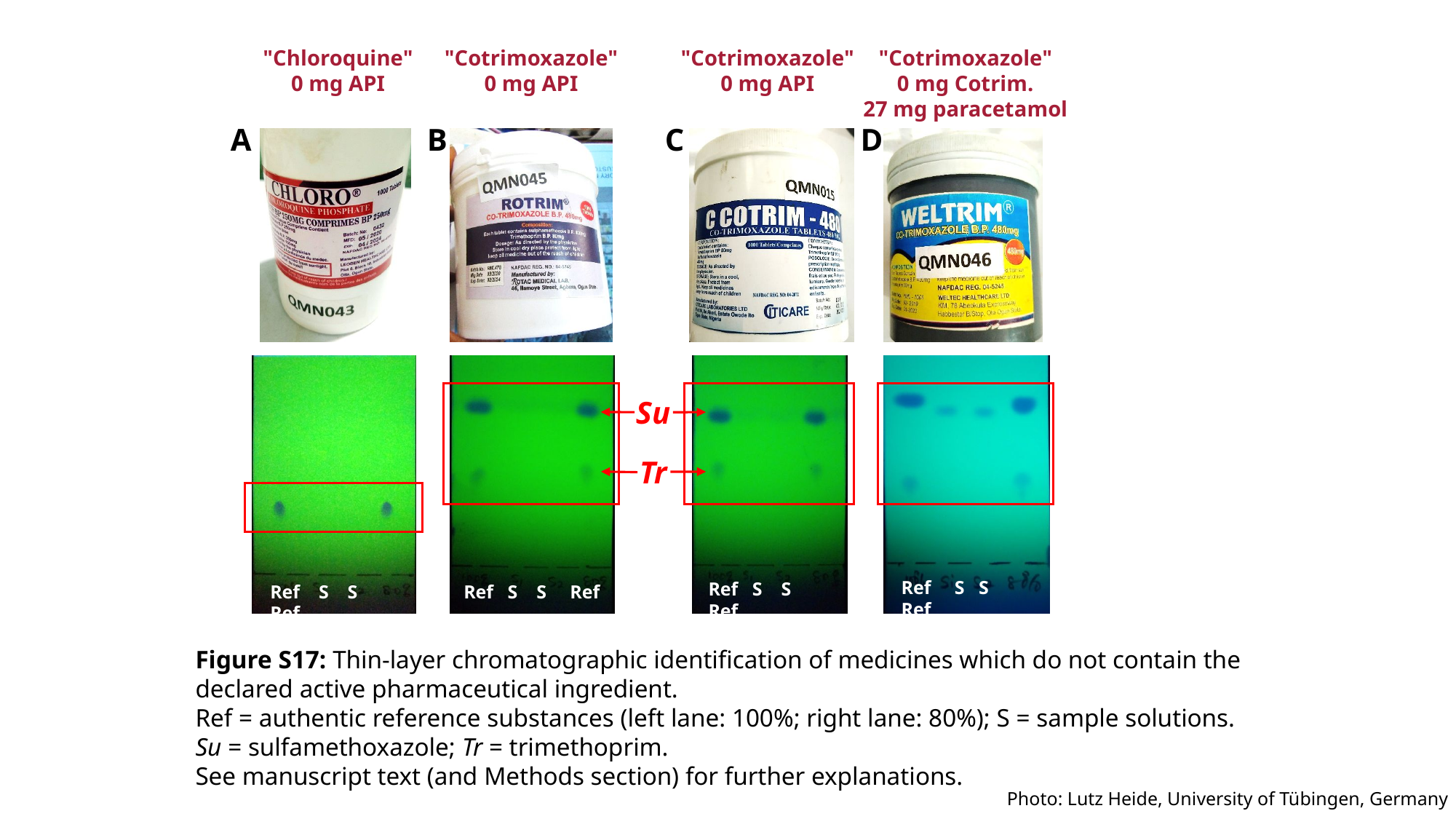

"Chloroquine"
0 mg API
"Cotrimoxazole"
0 mg API
"Cotrimoxazole"
0 mg API
"Cotrimoxazole"
0 mg Cotrim.
27 mg paracetamol
A
B
C
D
Ref S S Ref
Ref S S Ref
Ref S S Ref
Ref S S Ref
Su
Tr
Figure S17: Thin-layer chromatographic identification of medicines which do not contain the declared active pharmaceutical ingredient.
Ref = authentic reference substances (left lane: 100%; right lane: 80%); S = sample solutions.
Su = sulfamethoxazole; Tr = trimethoprim.
See manuscript text (and Methods section) for further explanations.
Photo: Lutz Heide, University of Tübingen, Germany

## Slide 18
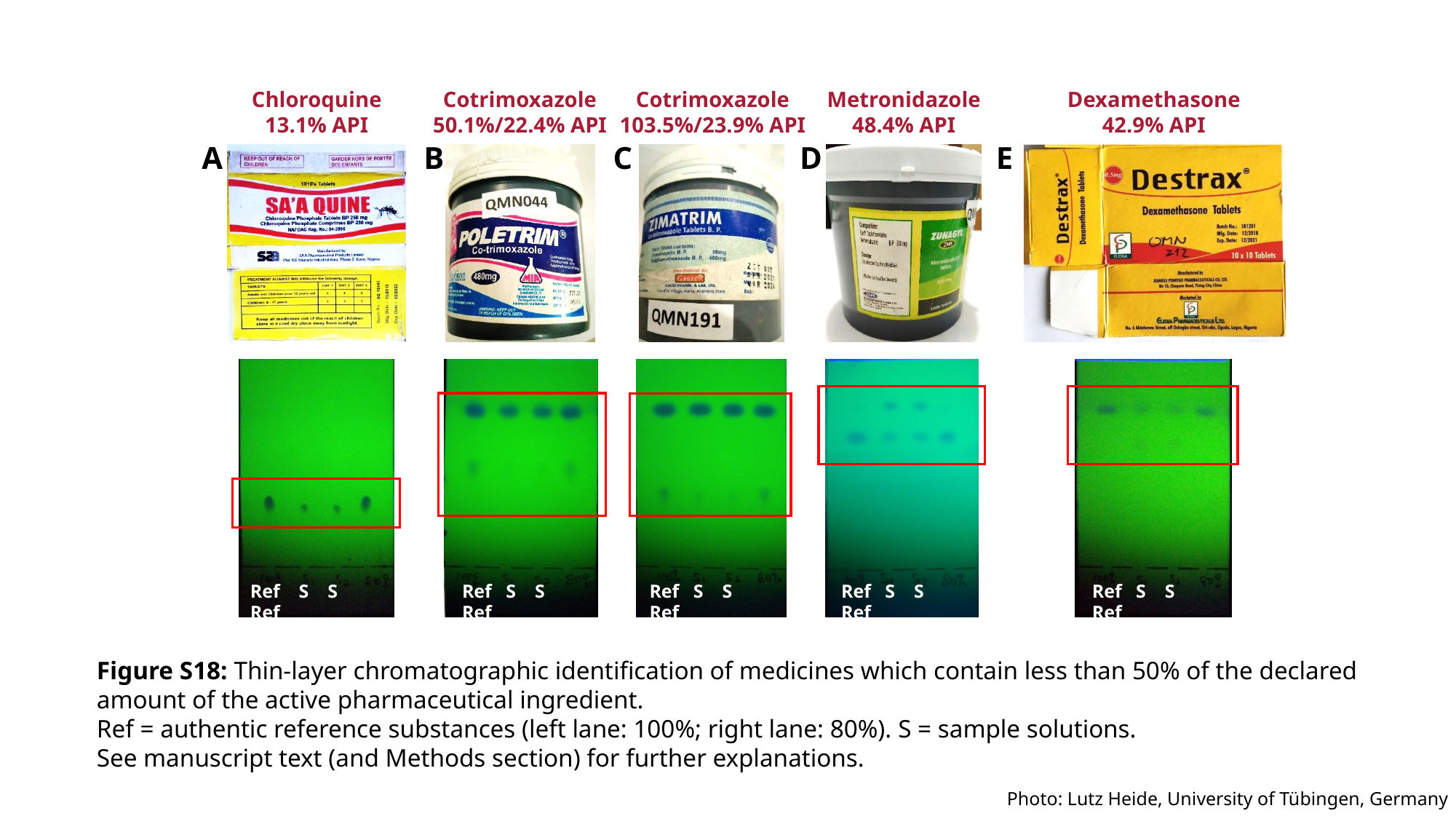

Chloroquine
13.1% API
Cotrimoxazole 50.1%/22.4% API
Cotrimoxazole 103.5%/23.9% API
Metronidazole
48.4% API
Dexamethasone
42.9% API
A
B
C
D
E
Ref S S Ref
Ref S S Ref
Ref S S Ref
Ref S S Ref
Ref S S Ref
Figure S18: Thin-layer chromatographic identification of medicines which contain less than 50% of the declared amount of the active pharmaceutical ingredient.
Ref = authentic reference substances (left lane: 100%; right lane: 80%). S = sample solutions.
See manuscript text (and Methods section) for further explanations.
Photo: Lutz Heide, University of Tübingen, Germany

## Slide 19
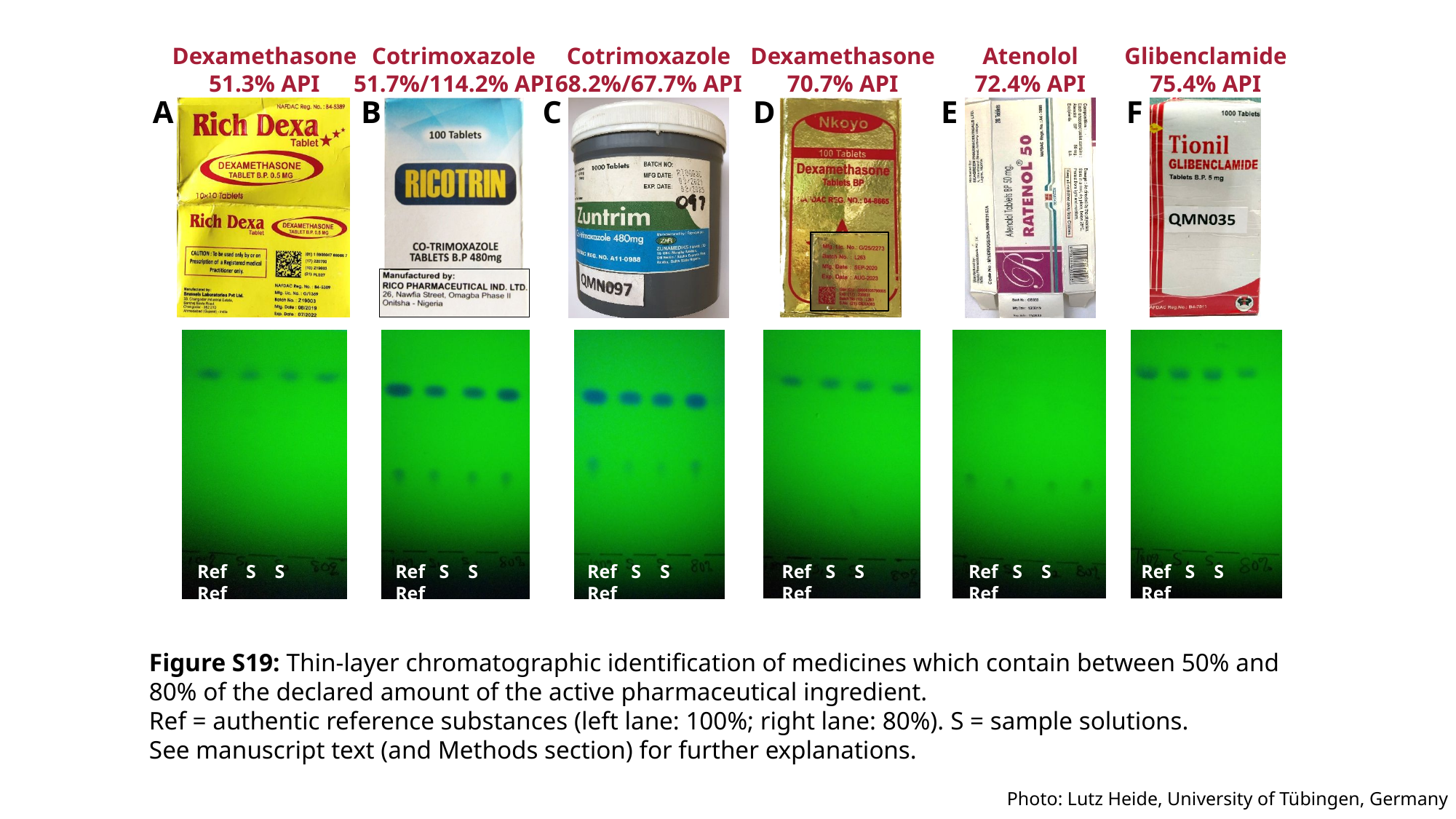

Dexamethasone
51.3% API
Cotrimoxazole
51.7%/114.2% API
Cotrimoxazole
68.2%/67.7% API
Dexamethasone
70.7% API
Atenolol
72.4% API
Glibenclamide
75.4% API
A
B
C
D
E
F
Ref S S Ref
Ref S S Ref
Ref S S Ref
Ref S S Ref
Ref S S Ref
Ref S S Ref
Figure S19: Thin-layer chromatographic identification of medicines which contain between 50% and 80% of the declared amount of the active pharmaceutical ingredient.
Ref = authentic reference substances (left lane: 100%; right lane: 80%). S = sample solutions.
See manuscript text (and Methods section) for further explanations.
Photo: Lutz Heide, University of Tübingen, Germany

## Slide 20
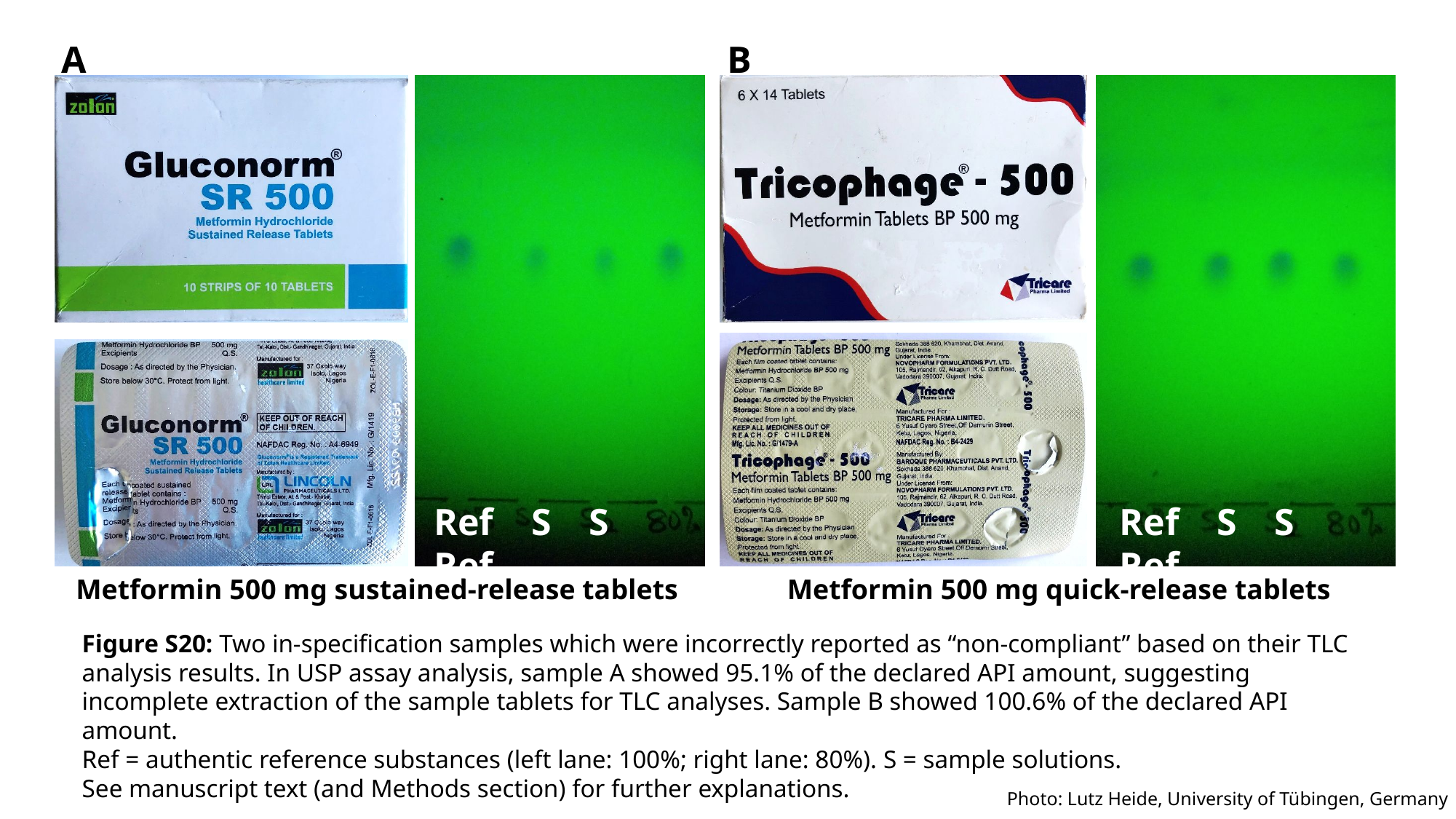

A
B
Ref S S Ref
Ref S S Ref
Metformin 500 mg sustained-release tablets
Metformin 500 mg quick-release tablets
Figure S20: Two in-specification samples which were incorrectly reported as “non-compliant” based on their TLC analysis results. In USP assay analysis, sample A showed 95.1% of the declared API amount, suggesting incomplete extraction of the sample tablets for TLC analyses. Sample B showed 100.6% of the declared API amount.
Ref = authentic reference substances (left lane: 100%; right lane: 80%). S = sample solutions.
See manuscript text (and Methods section) for further explanations.
Photo: Lutz Heide, University of Tübingen, Germany

## Slide 21
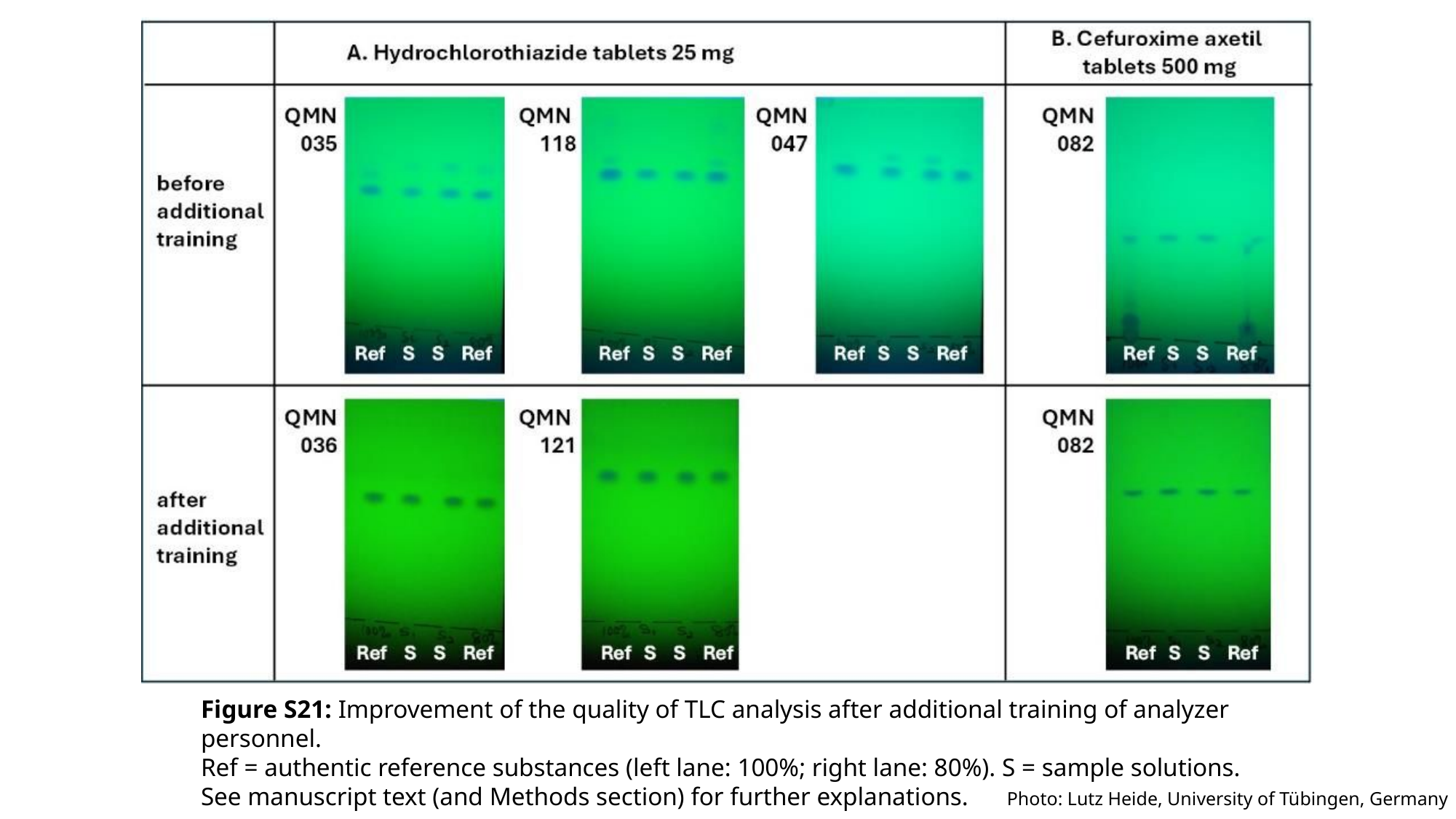

Figure S21: Improvement of the quality of TLC analysis after additional training of analyzer personnel.
Ref = authentic reference substances (left lane: 100%; right lane: 80%). S = sample solutions.
See manuscript text (and Methods section) for further explanations.
Photo: Lutz Heide, University of Tübingen, Germany
