# Supplementary material for: Screening for substandard and falsified medicines in Nigeria using visual inspection and GPHF-Minilab analysis: lessons learnt for future training of health workers and pharmacy personnel
Source: J Pharm Policy Pract. 2024 Dec 9;17(1):2432471. doi: 10.1080/20523211.2024.2432471 (PMC11632929; doi:10.1080/20523211.2024.2432471)
Supplement: Supplementary_Table_S1_Overview_of_medicines_investigated_Revised.pdf [file JPPP_A_2432471_SM8525.pdf]

Supplementary Table S1. Overview of medicines investigated in this study

No.	Active pharmaceutical ingredient (API)	Dosage form	Total number of samples	Number of different brands	Number of different batches	Number of samples investigated by:					
						Assay analysis according to USP 42	Dissolution analysis according to USP 42	Visual inspection	Simplified disintegration testing	TLC analysis with visual evaluation	TLC image analysis with TLCyzer app
1	Atenolol	tablet	14	7	12	14	14	14	12	12	12
2	Ceftriaxone	powder for injection	23	17	22	23	0	23	0	17	17
3	Cefuroxime axetil	tablet	24	13	18	24	24	24	24	24	24
4	Chloroquine	tablet	16	11	13	16	16	16	16	16	15
5	Ciprofloxacin	tablet	26	23	26	26	25	25	25	25	25
6	Dexamethasone	tablet	22	13	20	22	22	22	22	22	22
7	Fluconazole	capsule or tablet	22	11	17	22	15	22	1	0	0
8	Furosemide	tablet	14	7	12	14	14	14	14	14	13
9	Glibenclamide	tablet	19	10	16	19	19	19	19	19	19
10	Hydrochlorothiazide	tablet	16	7	14	16	16	16	15	15	15
11	Metformin hydrochloride	tablet	22	16	22	22	22	22	22	22	21
12	Metronidazole	tablet	25	12	24	25	25	25	25	25	25
13	Cotrimoxazole	tablet	17	13	17	17	17	17	17	17	14
	**Total**		**260**	**160**	**233**	**260**	**229**	**259**	**212**	**228**	**222**

All 260 samples were investigated by compendial assay analysis. Ceftriaxone injections were excluded from compendial dissolution testing and from simplified disintegration testing since these tests only apply for solid oral dosage forms. The seven fluconazole samples collected as capsules were excluded from dissolution testing since no dissolution method is described for them in USP. For one ciprofloxacin sample not enough dosage units were available for dissolution testing. Disintegration testing and TLC analysis of the fluconazole samples could not be completed in the time available for this study due to the COVID-19 pandemic and to civil unrest in Nigeria. The same applied to the TLC analysis of six ceftriaxone, two atenolol, one ciprofloxacin and one hydrochlorothiazide sample. Furthermore, in the quantitative evaluation of TLC results by image analysis, one “chloroquine” and three “cotrimoxazole” samples were excluded since they were falsified and did not show spots of the declared API(s) which could have been evaluated; in addition, one sustained-release metformin sample was excluded since it showed incomplete extraction of the API in TLC analysis (see Results section), therefore quantitative evaluation of the TLC spot of this sample by image analysis was not meaningful.
