# Supplementary material for: Screening for substandard and falsified medicines in Nigeria using visual inspection and GPHF-Minilab analysis: lessons learnt for future training of health workers and pharmacy personnel
Source: J Pharm Policy Pract. 2024 Dec 9;17(1):2432471. doi: 10.1080/20523211.2024.2432471 (PMC11632929; doi:10.1080/20523211.2024.2432471)
Supplement: Supplementary_Table_S2_Summary of the results of compendial assay analysis_Revised.pdf [file JPPP_A_2432471_SM8524.pdf]

Supplementary Table S2. Summary of the results of compendial assay analysis

No.	Active pharmaceutical ingredient (API)	Dosage form	Total number of samples	Falsified, not containing the declared API	API detected, but <50% of declared amount	API content from 50% to <80% of declared amount	API content ≥80% but lower than USP threshold	API content higher than USP threshold	API content within USP specifications
1	Atenolol	tablet	14	0	0	1	0	0	13
2	Ceftriaxone	powder for injection	23	0	0	0	0	0	23
3	Cefuroxime axetil	tablet	24	0	0	0	2	0	22
4	Chloroquine	tablet	16	1	1	0	1	1	12
5	Ciprofloxacin	tablet	26	0	0	0	5	0	21
6	Dexamethasone	tablet	22	0	1	7	12	0	2
7	Fluconazole	capsule or tablet	22	0	0	1	0	0	21
8	Furosemide	tablet	14	0	0	0	0	0	14
9	Glibenclamide	tablet	19	0	0	1	3	0	15
10	Hydrochloro-thiazide	tablet	16	0	0	0	1	0	15
11	Metformin hydrochloride	tablet	22	0	0	0	1	0	21
12	Metronidazole	tablet	25	0	1	0	1	0	23
13	Cotrimoxazole	tablet	17	3	2	2	0	0	10
	**Total**		**260**	**4**	**5**	**12**	**26**	**1**	**212**

Detailed compendial analysis results of all samples are published in Gabel et al. (2024) Am J Trop Med Hyg 111: 179-195.
